# Intravacuolar persistence in neutrophils facilitates *Listeria monocytogenes* spread to co-cultured cells

**DOI:** 10.1128/mbio.02700-24

**Published:** 2025-03-11

**Authors:** Stefano Bagatella, Camille Monney, Natascha Gross, Véronique Bernier Gosselin, Gertraud Schüpbach-Regula, Andrew Hemphill, Anna Oevermann

**Affiliations:** 1 Division of Neurological Sciences, NeuroCenter, Vetsuisse Faculty, University of Bern, Bern, Switzerland; 2 Graduate School for Cellular and Biomedical Sciences, University of Bern 27210 , Bern, Switzerland; 3 Clinic for Ruminants, Vetsuisse Faculty, University of Bern, Bern, Switzerland; 4 Veterinary Public Health Institute, Vetsuisse Faculty, University of Bern, Bern, Switzerland; 5 Institute of Parasitology, Vetsuisse Faculty, University of Bern, Bern, Switzerland; The University of Edinburgh, Edinburgh, United Kingdom

**Keywords:** listeriosis, neutrophils, bacterial resilience, vacuoles, reinfection, VBNC, resuscitation, spread

## Abstract

The bacterium *Listeria monocytogenes* (*Lm*) causes listeriosis in humans and ruminants. Acute lesions are predominantly infiltrated by polymorphonuclear neutrophils (PMNs), considered to be the efficient bactericidal arm of innate immunity. However, recent evidence suggests that PMNs cannot achieve antilisterial sterilizing immunity and that *Lm* may persist within PMNs. Despite this, interactions between PMNs and *Lm* remain poorly understood. In this study, we characterized the listericidal activity and interaction dynamics of bovine PMNs with *Lm ex vivo*. Phagocytosed *Lm* failed to escape into the PMN cytosol and was primarily targeted by phagolysosomal mechanisms. However, PMNs enabled prolonged intravacuolar survival of a resilient *Lm* subpopulation, largely as viable but non-culturable (VBNC) bacteria. This resilient *Lm* population could spread from PMNs to a cell line, resuscitate, and complete its canonical life cycle, thereby perpetuating the infection. Therefore, we identify PMNs as a mobile niche for *Lm* survival and provide evidence that PMNs harbor VBNC bacteria, potentially facilitating *Lm* dissemination within the host.

**IMPORTANCE:**

*Listeria monocytogenes* (*Lm*) is a significant foodborne pathogen responsible for high hospitalization rates in humans, especially vulnerable groups such as the elderly, pregnant women, and immunocompromised individuals. In animals like ruminants, *Lm* infection leads to severe disease manifestations, notably brainstem encephalitis. This study uncovers a novel mechanism by which bovine neutrophils (PMNs) harbor *Lm* in a viable but non-culturable (VBNC) state, enabling the bacteria to hide in the host. PMNs, traditionally viewed as bacteria killers, may serve as Trojan horses, allowing *Lm* to persist and spread within the host. This discovery has broad implications for understanding *Lm*'s persistence, its role in recurrent infections, and the development of new therapeutic strategies targeting VBNC forms of *Lm* to improve treatment outcomes and disease control.

## INTRODUCTION

*Listeria monocytogenes* (*Lm*) is a gram-positive bacterial saprophyte that, when ingested, can cause potentially fatal infections in humans and animals (1, 2). Listeriosis, the resulting disease, is associated with various clinical manifestations including gastroenteritis, septicemia, fetomaternal, and central nervous system (CNS) disease (neurolisteriosis) (2, 3). Among foodborne pathogens, *Lm* is responsible for the highest hospitalization rates in human patients and poses a significant threat to susceptible individuals such as infants, the elderly, pregnant women, and immunocompromised patients (1, 4, 5). Ruminants are the most commonly affected animal species, with listeriosis mainly manifesting as brainstem encephalitis (rhombencephalitis), which also occurs in humans (2, 6). Listeriosis in ruminants not only leads to livestock losses but is also a public health concern due to the potential transmission of *Lm* through contaminated animal products (6–8).

Successful host infection by *Lm* depends on establishing an intracellular life cycle within professional phagocytes and non-phagocytic cells (reviewed in reference 9). Following internalization or phagocytosis, *Lm* escapes from the primary vacuole through lysis, primarily mediated by the pore-forming hemolysin, listeriolysin-O (LLO), before the vacuole/phagosome fuses with a lysosome (10, 11). The released bacteria replicate in the cytosol and hijack the host cell actin, via the ActA protein (12, 13), to avoid xenophagic targeting (14, 15) and to spread to neighboring cells through polar actin polymerization (so-called actin tails) (16). In the new host cell, *Lm* is surrounded by a double-membrane vacuole, which *Lm* lyses again to continue its propagation (9).

The innate immune response is critical for *Lm* clearance, as early bacterial killing and containment at infection sites depend on monocytes/macrophages (17–19) and polymorphonuclear neutrophils (PMNs) (20–24). PMNs are the first blood-borne cells to accumulate in *Lm-*infected tissues, essential for the initial bacterial control through phagocytosis and reactive oxygen species (ROS) production (25, 26). In ruminants, PMNs are attracted by infected microglia (27) in the acute stage of listerial rhombencephalitis, forming parenchymal microabscesses (2, 6, 28) that contain high numbers of phagocytosed bacteria (29). However, bacterial load in these microabscesses only decreases with the infiltration of CD3^+^ T cells during later infection stages (30), consistent with murine infection studies that highlight the role of CD4+ and CD8+ T cells in achieving sterilizing immunity against *Lm* (31–33). These observations suggest that PMNs may not efficiently clear *Lm* during infection. In addition, transmission electron microscopy (TEM) studies in ruminants have identified morphologically intact *Lm* inside intra-axonal PMNs (28), questioning the bactericidal efficiency of PMNs and suggesting that they may provide a niche for bacterial survival.

In this study, we aimed to elucidate the interaction and fate of *Lm* in PMNs following phagocytosis by investigating the infection dynamics of *Lm* in bovine PMNs *ex vivo*. We provide evidence that phagocytosed *Lm* does not escape the vacuoles of bovine PMNs. Although most bacteria are killed, a subset of live *Lm* persists within PMNs vacuoles. Some *Lm* are viable and culturable, but a major proportion persists as viable but non-culturable (VBNC) bacteria. These persisting bacteria can be taken up by a bovine macrophage cell line upon PMN loss of integrity, resuscitate in these cells, and complete their canonical life cycle. Thus, our data identify PMNs as a novel mobile niche for *Lm* survival and cellular transmission, potentially facilitating dissemination within the host.

## RESULTS

### Bovine PMNs are unable to sterilize extracellular and intracellular *Lm*, regardless of multiplicity of infection (MOI) or opsonization

We first evaluated the listericidal capacity of bovine PMNs against the extra- and intra-cellular population of a clinically relevant *Lm* strain isolated from a cow with neurolisteriosis (JF5203, https://www.ncbi.nlm.nih.gov/nuccore/NZ_LT985474.1; referred to as wild-type (WT)-*Lm*). To this end, we performed killing and gentamicin assays, in which colony-forming units (CFU) of WT-*Lm* co-incubated with PMNs at multiplicities of infection (MOIs) of 0.1 and 5 were quantified.

In killing assays, CFUs of WT-*Lm* co-incubated with PMNs at a MOI of 0.1 decreased by 30% within the first 10 min compared with controls without PMNs (Fig. S1A and C). However, CFU counts then stabilized and increased again after 5 h. At MOI of 5, CFU reduction by PMNs was 45% at early time points compared with controls without PMNs (Fig. S1B and C), but this effect was not statistically significant (Fig. S1B). Similar to the lower MOI, *Lm* numbers increased at later time points (5 h, 24 h) (Fig. S1A and B). Serum opsonization increased CFU reduction by PMNs at early time points (Fig. S1D), but PMNs were still unable to prevent bacterial replication at later time points (Fig. S1D and S2A). Thus, although PMNs can kill a large fraction of *Lm* and serum opsonization enhances bacterial killing, they are unable to sterilize *Lm*.

**Fig 1:**
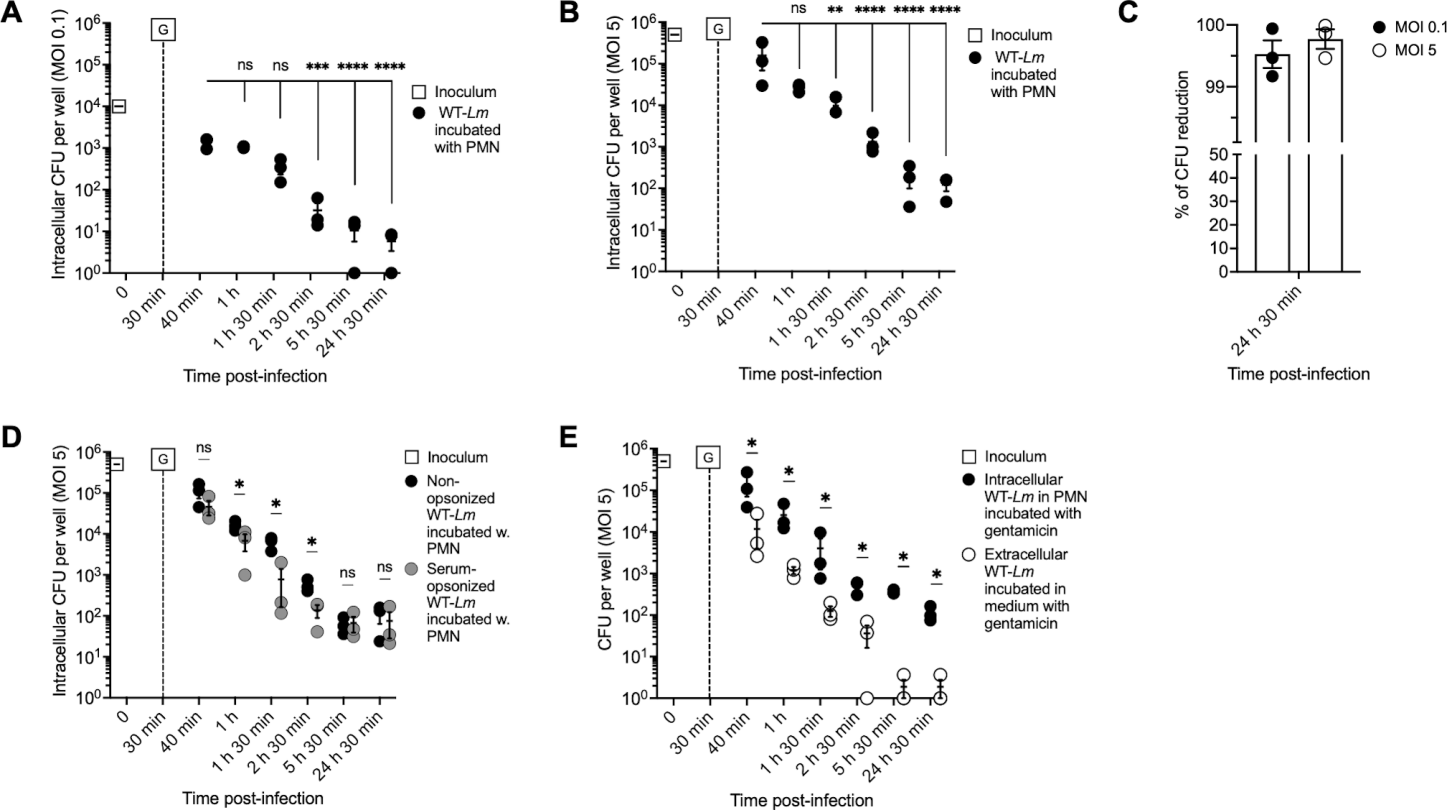
Bovine PMNs fail to efficiently sterilize intracellular *Lm*. (**A, B**) Gentamicin protection assay enumerating CFU of phagocytosed WT-*Lm* incubated with PMNs at MOI of 0.1 (**A**) or 5 (**B**). Gentamicin (G) was added to the medium 30 min p.i. CFU numbers are compared with the first incubation time point (40 min p.i.). (**C**) Percentage of intracellular CFU reduction by PMNs at 24 h 30 min p.i. in the gentamicin assay (MOI 0.1: black; MOI 5: white). Values indicate CFU reduction at 24 h 30 min p.i. compared with the first time point (40 min p.i.). (**D**) Gentamicin protection assay of PMNs infected with non-opsonized WT-*Lm* (black) and serum-opsonized WT-*Lm* (gray) at MOI of 5. Gentamicin (G) was added to the medium at 30 min p.i. in all assays. (**E**) CFU of WT-*Lm* (MOI 5) incubated with PMNs in the gentamicin protection assay (black) compared with bacteria incubated in gentamicin-containing medium without PMNs (white). Gentamicin (G) was added to the medium 30 min p.i. Depending on the MOI used, CFU were normalized to an initial inoculum of 10^4^ CFU (MOI 0.1: A) or 5 × 10^5^ CFU (MOI 5: B, **D, E**). Data are expressed as the mean (±SEM) of CFU per well from three independent experiments performed in triplicate. Data were analyzed by one-way ANOVA followed by planned comparisons (**A, B**) or Kruskal-Wallis test (**D, E**). * = *P* < 0.05; ** = *P* < 0.01; *** = *P* < 0.001; **** = *P* < 0.0001; ns = not significant.

To assess PMN killing efficiency against phagocytosed *Lm*, we performed gentamicin protection assays. Gentamicin, an aminoglycoside antibiotic that does not effectively penetrate into PMNs (34), was added after 30 min of phagocytosis to kill non-internalized *Lm*. At MOIs of 0.1 and 5, PMNs progressively reduced intracellular CFU counts (Fig. 1A and B), indicating effective intraneutrophilic bactericidal mechanisms up to late time points. Despite this, less than 1% of bacteria were culturable at 24 h 30 min post-infection (p.i.) compared with 40 min p.i. (Fig. 1C), with low numbers of culturable bacteria still recovered at later time points, particularly at MOI 5 (Fig. 1A and B). Serum opsonization slightly enhanced the killing of intraneutrophilic bacteria but had a minor impact on bacterial killing in comparison to previous publications (35, 36) and did not achieve sterilization (Fig. 1D; Fig. S2B). This low impact can be explained by PMN priming by centrifugation for adherence and synchronization of infection that may enhance bacterial phagocytosis and killing in the absence of opsonins (37). The reduction in intracellular CFU was not due to a direct bactericidal effect of fresh serum components, as both opsonized and non-opsonized *Lm* incubated without PMNs showed similar growth curves (Fig. S2C).

To ensure the reduction in intracellular bacteria was not due to gentamicin penetration into PMNs, we compared the dynamics of bacteria co-cultured with PMNs to those of extracellular bacteria incubated without PMNs in the presence of gentamicin. Although bacterial numbers decreased over time in both conditions, CFU counts were consistently higher when *Lm* was incubated with PMNs (Fig. 1E). Notably, although extracellular *Lm* was nearly sterilized after 5 h 30 min, CFUs continued to be recovered from intraneutrophilic *Lm*, indicating that PMNs sheltered intracellular *Lm* from extracellular gentamicin. Since bacterial dynamics were similar between tested MOIs, subsequent experiments were performed using an MOI of 5. The effect of serum opsonization on *Lm* viability and intraneutrophilic dynamics was further evaluated in selected experiments (see below and Fig. S2D through K).

### Viable intraneutrophilic *Lm* remain stable over time and are underestimated by CFU quantification

**Fig 2:**
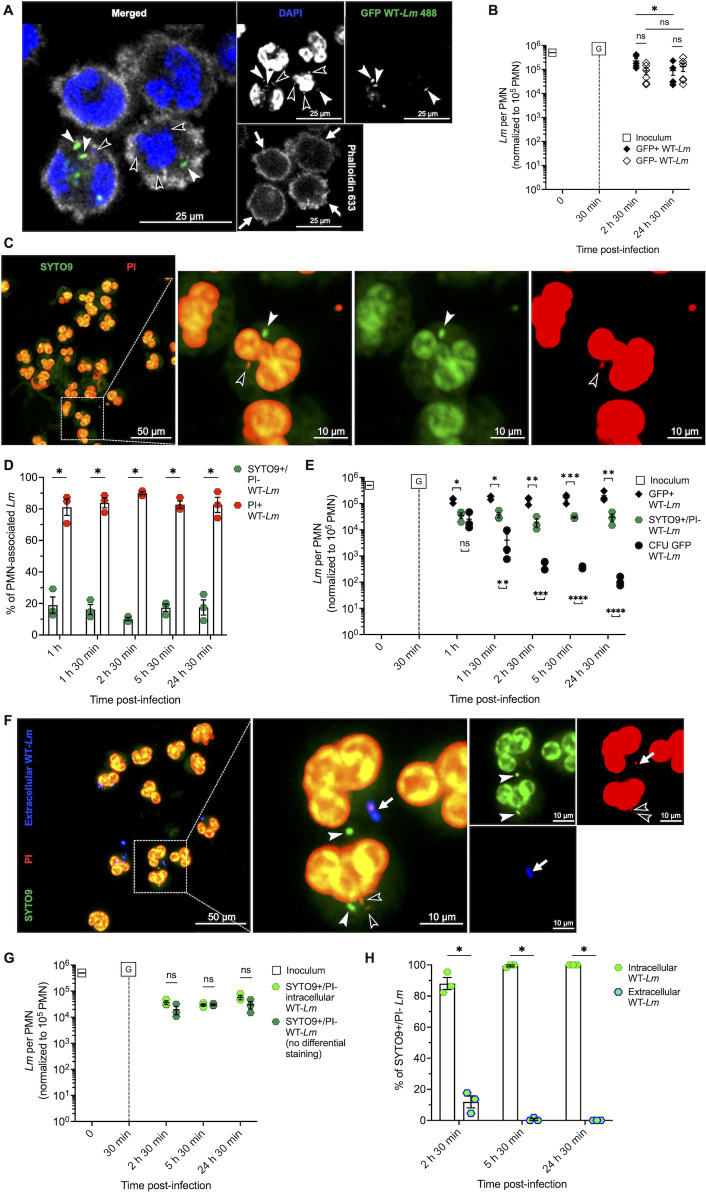
The number of viable intraneutrophilic *Lm* exceeds the number of CFU recovered from PMNs. (**A**) Representative image of PMNs infected with GFP-expressing WT-*Lm* at 2 h 30 min p.i. in a gentamicin protection assay. Nuclei are stained blue (DAPI) and actin is stained gray (phalloidin). Bacteria were considered intracellular if they were located within the PMN phalloidin ring (white arrows). Note the lack of actin polymerization of both GFP+ (white arrowheads) and GFP– (empty arrowheads) *Lm* within the PMN phalloidin ring, indicating bacterial intravacuolar confinement. (**B**) Number of intraneutrophilic GFP+ (black) and GFP– (white) WT-*Lm*. Data are presented as mean (±SEM) of *Lm* per PMN (normalized to 10^5^ PMNs) obtained from six independent experiments (in which 10 independent FOVs were analyzed for each replicate at each time point). Gentamicin (G) was added to the medium 30 min p.i. (**C-H**) Viability of phagocytosed WT-*Lm* in bovine PMNs (gentamicin protection assay). (**C**) Representative images of the BacLight viability assay at 2 h 30 min p.i. Viable *Lm* stain green with SYTO9 and not with PI (white arrowhead), whereas dead/permeabilized *Lm* stain red with PI (empty arrowhead) and nuclei of permeabilized PMNs stain with both markers. (**D**) Percentage of viable, SYTO9+/PI– (green) and non-viable, PI+ (red) *Lm* associated with bovine PMNs. Data are expressed as mean ±SEM from three independent experiments (in which 10 independent FOVs were analyzed for each replicate at each time point). (**E**) Comparison between estimated viable SYTO9+/PI– *Lm* (green), GFP+ *Lm* (black diamonds) and CFU (black circles). CFU data are from Fig. 1E, as these experiments were performed in parallel with those that generated the GFP+ *Lm* data shown here. Data are expressed as mean (±SEM) of *Lm* per 10^5^ PMNs from three independent experiments (performed in triplicate for CFU; 10 independent FOVs per experiment and time point for GFP+ and SYTO9+/PI– *Lm*). Gentamicin (G) was added to the medium at 30 min p.i. (**F**) Representative MAX intensity projections of Z-stacks from the BacLight viability assay combined with differential intra-/extracellular *Lm* staining at 2 h 30 min p.i. Viable *Lm* are shown in green (white arrowhead), dead *Lm* are shown in red (empty arrowhead), and extracellular *Lm* are shown in blue (white arrow). (**G**) Comparison between viable intracellular *Lm* (light green) and viable PMN-associated *Lm* counted without the differential intra-/extracellular staining (from Fig. 2E, dark green). Data are expressed as mean (±SEM) of *Lm* per 10^5^ PMNs from three independent experiments, in which 10 independent FOVs (for SYTO9+/PI– WT-*Lm* without differential staining) or five independent FOVs (for SYTO9+/PI– intracellular WT-*Lm*) were analyzed for each experiment at each time point. Gentamicin (G) was added to the medium at 30 min p.i. (**H**) Percentage of PMN-associated intracellular viable WT-*Lm* (green) compared with PMN-associated extracellular viable bacteria (green with blue border). Data are expressed as mean (±SEM) from three independent experiments (in which five independent FOVs were analyzed for each replicate at each time point). Data were analyzed by Kruskal-Wallis test (**B, D, G, H**) or one-way ANOVA followed by planned comparisons (**E**). * = *P* < 0.05; ** = *P* < 0.01; *** = *P* < 0.001; **** = *P* < 0.0001; ns = not significant.

Since CFU analysis indicated that *Lm* survives in low numbers within bovine PMNs, we aimed to further characterize the intraneutrophilic lifestyle of *Lm*. We co-incubated GFP-expressing *Lm* with bovine PMNs in a gentamicin protection assay and quantified phagocytosed bacteria using confocal immunofluorescence analysis (Fig. 2A). Phalloidin staining of infected PMNs revealed a submembranous ring of actin filaments (phalloidin ring) (Fig. 2A) used to differentiate extracellular from intracellular *Lm*. Intracellular GFP+ and GFP– bacteria were enumerated, with GFP expression initially used as a proxy for bacterial viability (38, 39). To allow an approximate comparison between CFU and microscopic bacterial counts, we normalized the average microscopic counts of bacteria per PMN to 10^5^ PMN (see Materials and Methods: “Immunofluorescence assays”). Surprisingly, the decrease in GFP+ bacteria from 2 h 30 min to 24 h 30 min p.i. was minimal compared with the CFU decrease (Fig. 2B). In subsequent experiments, the number of GFP+ intracellular bacteria showed remarkably different dynamics compared with CFUs, remaining relatively constant and 1–3 orders of magnitude higher than CFU throughout the experiment (Fig. 2E). This led us to consider that the majority of the GFP+ population might represent viable but non-culturable (VBNC) *Lm* (40). To more reliably assess bacterial viability, we used the BacLight assay (41) to differentiate total bacteria (SYTO9+) from damaged/dead bacteria (PI+) in a gentamicin protection assays (Fig. 2C). Viable/intact (SYTO9+/PI–) *Lm* consistently accounted for 10%–20% of PMN-associated bacteria (Fig. 2D). Although GFP+ bacteria overestimated viable *Lm* by approximately one order of magnitude, the dynamics of SYTO9+/PI– *Lm* was similar, remaining relatively constant and higher than CFU counts over time (Fig. 2E). These results confirm that CFU counts, which only detect culturable bacteria, underestimate the true number of viable intraneutrophilic *Lm*, supporting the presence of a significant VBNC *Lm* population within PMNs. The numbers of CFU and viable (SYTO9+/PI–) *Lm* were closest at 1 h p.i. before the subsequent decline in CFU counts (Fig. 2E), suggesting that resilient intraneutrophilic *Lm* begin switching to the VBNC state following 1 h post-phagocytosis, with the number of VBNC *Lm* progressively increasing over time.

To confirm the intraneutrophilic location of viable *Lm*, we performed Z-stack analysis of the BacLight assay, incorporating differential immunolabeling of extra- and intra-cellular bacteria with an antilisterial antibody (Fig. 2F). The number of viable intracellular (SYTO9+/PI–/*Lm*–) *Lm* was consistent with results obtained without differential intra-/extra-cellular *Lm* staining, confirming that most viable bacteria were indeed intracellular (Fig. 2G and H). Similar results were obtained using opsonized WT-*Lm* (Fig. S2D), with intracellular *Lm* viability unaffected by serum opsonization (Fig. S2E). Additionally, viable intracellular *Lm* identified by microscopy greatly outnumbered CFU counts in WT-*Lm*-infected human PMNs (Fig. S3A and B). The majority of viable bacteria were also intracellular (Fig. S3C), indicating that *Lm* can persist as VBNC in PMNs across different susceptible species.

### *Lm* are confined to heterogeneous single-membrane vacuoles in bovine PMNs

At all analyzed time points, phalloidin staining of bovine PMNs did not detect any *Lm*-associated actin polymerizations, such as actin clouds or actin tails, indicating that phagocytosed *Lm* remain confined to vacuolar compartments within PMNs (Fig. 2A). To confirm this, we performed a digitonin permeabilization assay (42), where extravacuolar *Lm* (both intracytosolic and extracellular) are stained following digitonin permeabilization of the PMN cell membrane, whereas intravacuolar bacteria remain unstained (Fig. S4A and B). The efficacy of permeabilization was verified by PI+ nuclear staining of all digitonin-permeabilized PMNs, whereas most non-permeabilized PMNs showed no PI-staining (Fig. S4C and D). Given the small cytoplasmic volume of bovine PMNs and the adherence of extracellular *Lm* to the PMN surface, distinguishing intra- from extra-cellular stained bacteria was challenging (Fig. S4A and B) and the number of stained *Lm* could not reliably quantify intracytosolic bacteria. Therefore, we compared the number of unstained *Lm* in permeabilized PMNs (intravacuolar *Lm*) with unpermeabilized PMNs (intracellular *Lm*) to determine the proportion of intravacuolar *Lm*. As shown in Fig. S4E, essentially no difference was observed between the number of intravacuolar *Lm* in permeabilized PMNs and intracellular *Lm* in unpermeabilized PMNs, confirming that phagocytosed *Lm* were trapped in vacuoles, preventing their escape into the cytosol.

**Fig 3:**
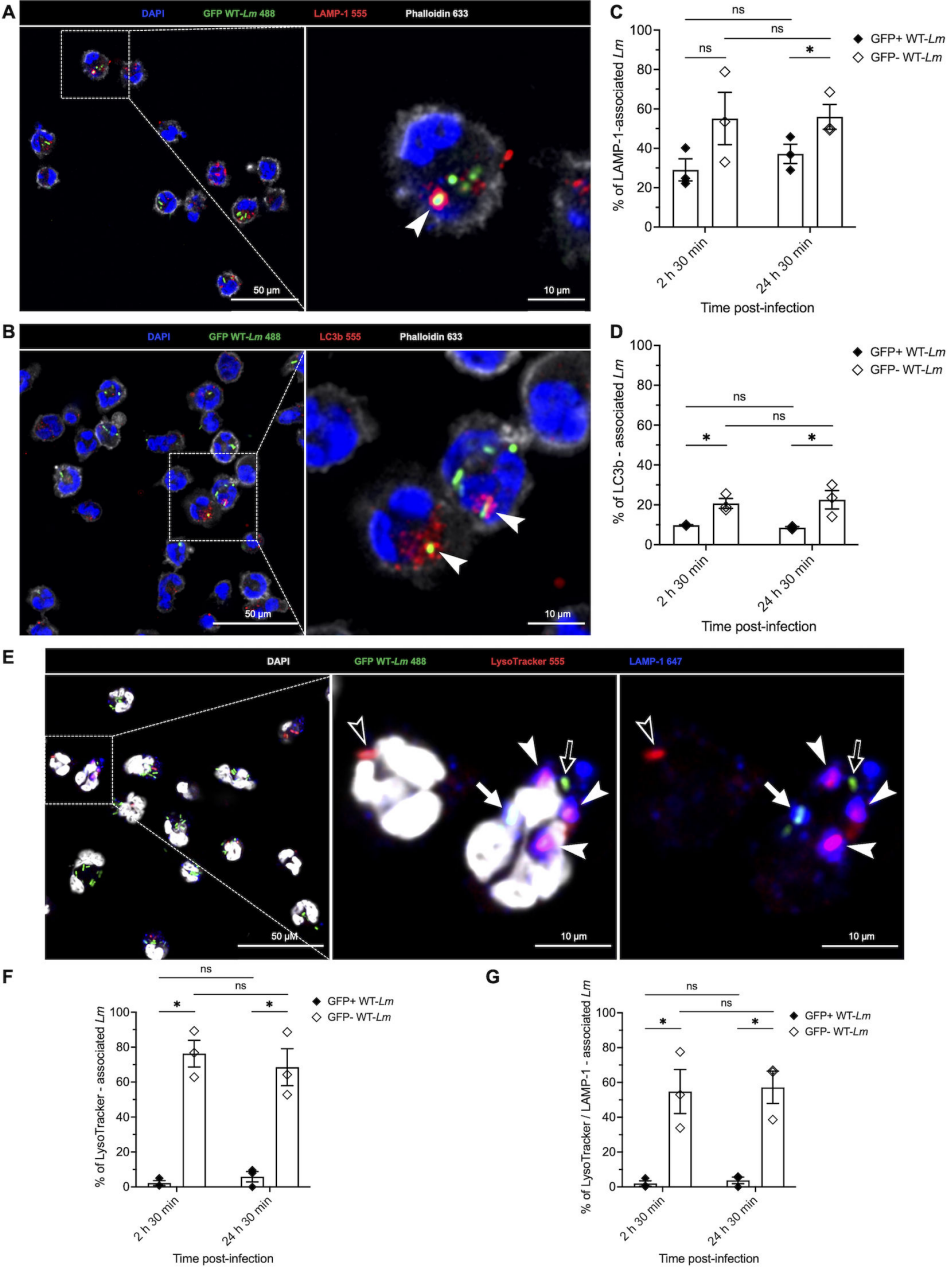
Intraneutrophilic *Lm* are sequestered in vacuoles with a heterogeneous phenotype. (**A, B**) Representative images of *Lm* association with LAMP-1 (**A**) or LC3b (**B**) at 2 h 30 min p.i. Both markers are shown in red and co-localization with GFP+ WT *Lm* (green) is indicated by white arrowheads. Nuclei are stained blue (DAPI), whereas actin is stained in gray (phalloidin). (**C, D**) Percentage of GFP+ (black diamonds) and GFP– (white diamonds) intracellular WT-*Lm* in association with LAMP-1 (**C**) or LC3b (**D**). Data are expressed as mean ±SEM from three independent experiments (in which 10 independent FOVs were analyzed per replicate and each time point). (**E**) Representative images of PMNs infected with GFP-expressing WT-*Lm* at 2 h 30 min p.i. Nuclei are stained with DAPI (gray), whereas *Lm*-containing LAMP-1-labeled vacuoles are stained in blue (white arrow), and acidic vacuoles stain positive with LysoTracker (red). Note that all acidic vacuoles contain GFP– *Lm* (empty arrowheads) and most acidic vacuoles co-localize with LAMP-1 (white arrowheads). Only one acidic vacuole is LAMP-1 negative (empty arrowhead). GFP+ bacteria (green) are located in non-acidic vacuoles (arrows) and do (white arrow) or do not (empty arrow) co-localize with LAMP-1. (**F, G**) Percentage of GFP+ (black diamonds) and GFP– (white diamonds) intracellular WT-*Lm* that are associated with LysoTracker only (**F**) or with both LysoTracker and LAMP-1 (**G**). Data are expressed as mean ±SEM from three independent experiments (in which 10 independent FOVs were analyzed per replicate and timepoint). Data were analyzed by Kruskal-Wallis test (**C, D, F, G**). * = *P* < 0.05; ns = not significant.

Intracellular persistence of *Lm* has been linked to its intravacuolar lifestyle, involving both phagosomal and autophagic pathways in various cell types (40, 43, 44). We investigated the involvement of these mechanisms in *Lm* persistence within bovine PMNs by evaluating the co-localization of phagocytosed *Lm* with LAMP-1 (Fig. 3A) and LC3b (Fig. 3B) vacuole markers. Throughout the experiment, 20%–45% of GFP+ *Lm* were associated with LAMP-1, with higher association rates for GFP– *Lm* (55%–80%) (Fig. 3C). Serum opsonization did not significantly increase *Lm* association with LAMP-1 (Fig. S2F). Only 10% of GFP+ and less than 30% of GFP– *Lm* were associated with LC3b (Fig. 3D).

Bacterial killing in PMN phagosomes correlates with an initial vacuolar alkalinization (favorable to the bactericidal action of granule proteins), followed by slow acidification favorable for lysosomal enzymes, whereas rapid acidification impairs bacterial killing (45). Since a significant number of *Lm* were associated with LAMP-1+ vacuoles, representing phagosomal compartments, we further characterized the pH of these *Lm*-containing vacuoles using LAMP-1 and an acidophilic dye (LysoTracker) staining (Fig. 3E). Less than 10% of GFP+ *Lm* localized to acidic compartments, whereas most GFP– bacteria (> 50%) localized to acidic vacuoles throughout the experiment (Fig. 3F). Similarly, fewer than 10% of GFP+ bacteria were positive for both LAMP-1 and LysoTracker, whereas 30%–80% of GFP– bacteria stained for both markers (Fig. 3G). Thus, GFP– bacteria preferentially, but not exclusively, localized to vacuoles displaying characteristics of mature phagosomes or phagolysosomes (i.e., acidic pH and LAMP-1+). Concurrent experiments performed in our laboratory (L. Tavares-Gomes et al., unpublished data) indicated that in contrast to results reported for other bacteria (38, 39), GFP is an unreliable viability indicator for *Lm*, as its signal is quenched at acidic pH without correlating to culturability or viability. Therefore, colocalization of GFP- bacteria within LysoTracker positive vacuoles confirms that bacteria become trapped in acidic vacuoles but provides no information on bacterial viability.

**Fig 4:**
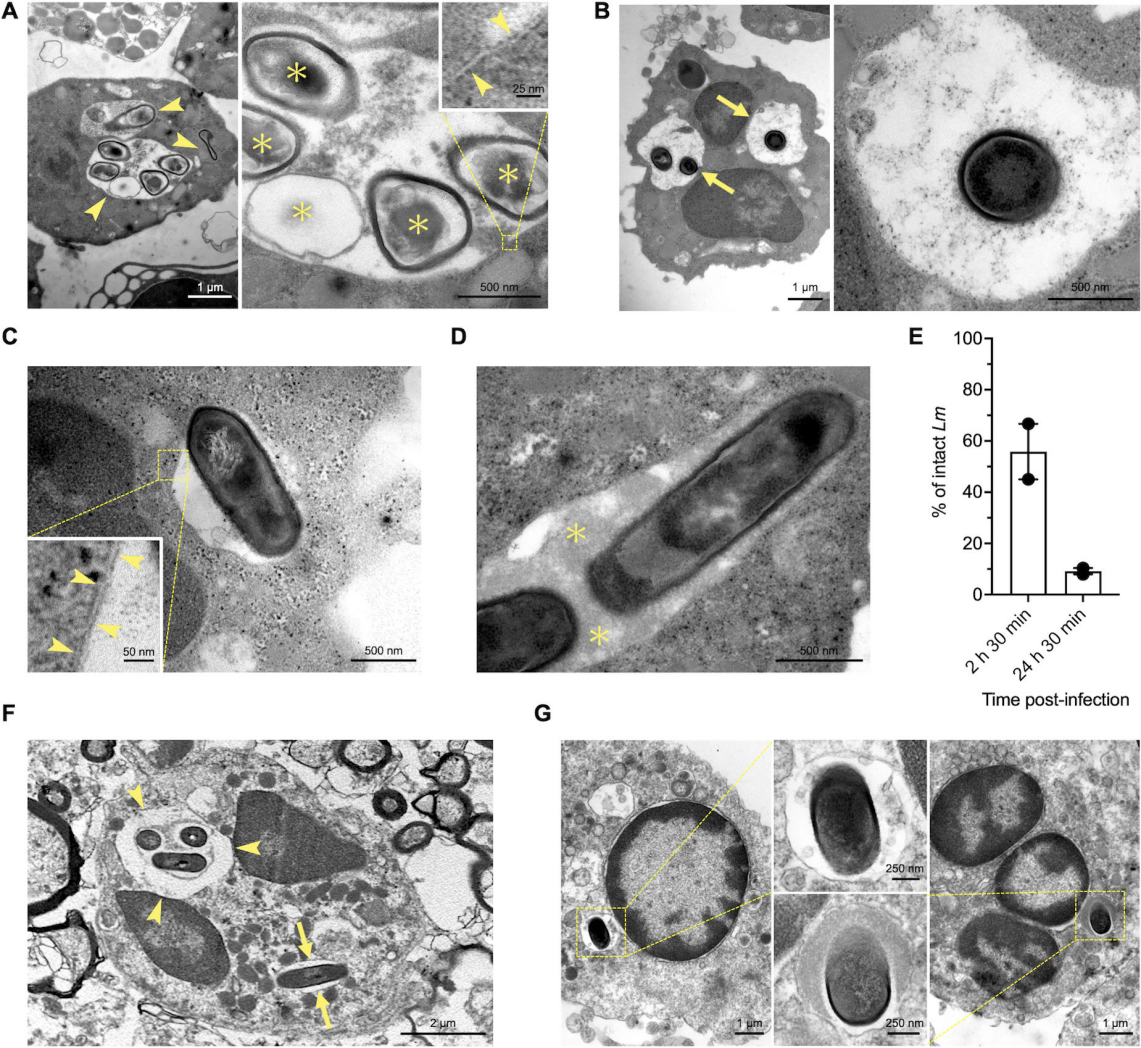
*Lm* are restricted to single-membrane vacuoles in bovine PMNs that recapitulate features of PMN vacuoles *in vivo*. Ultrastructural features of *Lm*-containing vacuoles in bovine PMNs infected *ex vivo* (**A–E**). (**A**) Spacious vacuoles containing damaged *Lm* (arrowheads, left panel, 24 h 30 min p.i.). Higher magnification of one of these vacuoles (right panel) shows central nucleoid and membrane material (asterisks) detached from the overlying electron-dense bacterial cell wall, indicating bacterial damage. The inset highlights the trilaminar appearance of the single vacuolar membrane (arrowheads). (**B**) Spacious vacuoles containing intact *Lm* (arrows, left panel, 24 h 30 min p.i.). The right panel shows a higher magnification of one of these vacuoles containing a morphologically intact bacterium (showing no separation between the cell wall and the underlying cell membrane and nucleoid material). (**C**) Tightly-fitting vacuole is largely devoid of intraluminal electron-dense material (2 h 30 min p.i.). The trilaminar aspect of the single vacuolar membrane (arrowheads) is shown in the inset. (**D**) Tightly fitting vacuole containing abundant intraluminal electron-dense material (asterisks, 2 h 30 min p.i.). (**E**) Percentage of morphologically intact intravacuolar *Lm* at 2 h 30 min p.i. and 24 h 30 min p.i. Data are the mean of two independent experiments ±SEM. Ultrastructural features of *Lm*-containing vacuoles in animals with clinical neurolisteriosis (**F and G**). (**F**) PMNs in a brainstem microabscess of a sheep with neurolisteriosis. A spacious vacuole containing three *Lm* (arrowheads) and a tightly fitting vacuole containing a single *Lm* (arrows) are observed. (**G**) PMNs in the meninges of a cotton-top tamarin (*Saguinus oedipus*) with listerial meningitis. Tightly-fitting vacuoles with a morphologically intact bacterium containing no (left) or abundant electron-dense material (right).

To further investigate *Lm*-containing vacuoles and the fate of *Lm* in PMNs, we performed transmission electron microscopy (TEM) of bovine PMNs infected *ex vivo* at 2 h 30 min and 24 h 30 min p.i. (Fig. 4A through E). Intraneutrophilic *Lm*, both damaged (Fig. 4A) and intact (Fig. 4B through D), were observed exclusively in single-membrane vacuoles, indicated by the trilaminar structure of the vacuolar membrane (Fig. 4A and C). These vacuoles were either spacious (Fig. 4A and B) or tightly fitting (Fig. 4C and D). Spacious vacuoles contained single or multiple bacteria (Fig. 4A and B), whereas tightly fitting vacuoles usually contained a single bacterial cell (Fig. 4C) or rarely two (Fig. 4D). Both vacuole types either contained electron-dense material (Fig. 4A and D) or were empty, except for the bacterium (Fig. 4B and C). We enumerated morphologically damaged (dead) and intact (viable) intravacuolar *Lm* to confirm our viability assay results. At 2 h 30 min p.i., approximately 45%–67% of *Lm* were intact with a continuous bacterial cell wall, whereas at 24 h 30 min p.i., 7.9%–10.3% of *Lm* remained intact (Fig. 4E). Thus, the percentage of viable bacteria was comparable with that observed in the BacLight assays. To determine whether *Lm*-containing PMN vacuoles displayed ultrastructural features similar to those observed *in vivo*, we screened cases of naturally infected animals with neurolisteriosis. Both spacious (Fig. 4F) and tightly fitting vacuoles (Fig. 4F and G) containing *Lm* were observed in intralesional PMNs, providing evidence that *Lm*-containing vacuoles of PMNs infected *ex vivo* recapitulate morphological features seen in naturally infected animals.

### *Lm* persistence in bovine PMNs is promoted by *hly*

**Fig 5:**
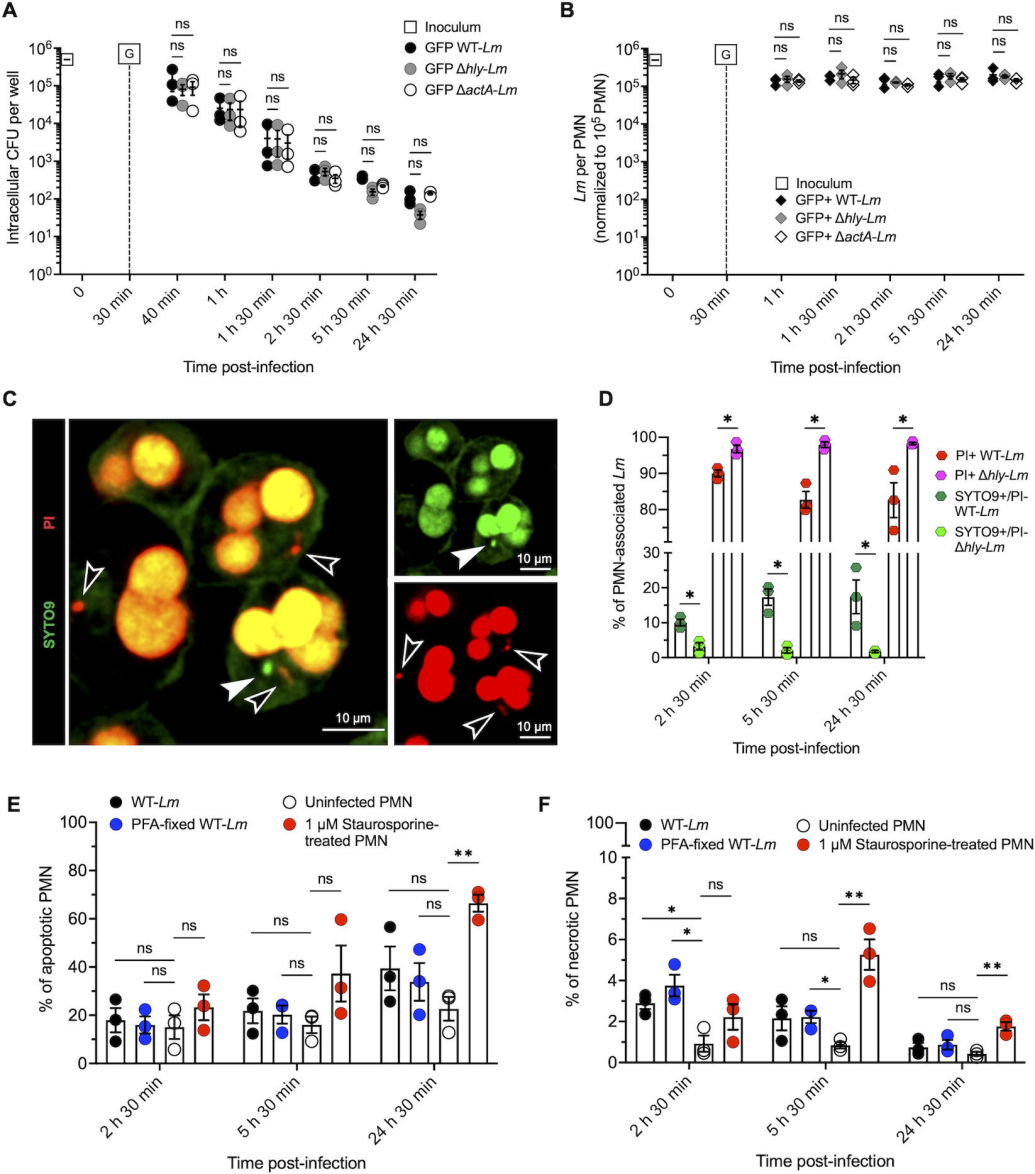
*hly* increases the ratio of persistent intraneutrophilic *Lm*, and *Lm* does not modulate PMN viability. (**A, B**) Intraneutrophilic dynamics of Δ*hly*- and Δ*actA-Lm* compared with WT. (**A**) Gentamicin protection assay comparing WT-*Lm* (black circles) with *Lm* deletion mutants deficient in LLO (Δ*hly*, gray circles) or ActA (Δ*actA*, white circles with black borders) (MOI 5). WT-*Lm* data are from Fig. 1I, as these experiments were performed in parallel. Gentamicin (G) was added to the medium at 30 min p.i. Data are expressed as mean (±SEM) of CFU per well from three independent experiments performed in triplicate, with CFU normalized to an initial inoculum of 5 × 10^5^ CFU. (**B**) Number of GFP+ intraneutrophilic WT-*Lm* (black), Δ*hly-Lm* (grey) and Δ*actA-Lm* (white) quantified by microscopy. GFP+ WT *Lm* data are from Fig. 2E, as these experiments were performed in parallel. Data are expressed as mean (±SEM) of *Lm* per 10^5^ PMN from three independent experiments (in which 20 independent FOVs were analyzed per replicate and time point). Gentamicin (G) was added to the medium at 30 min p.i. For each time point, the number of GFP+ WT *Lm* is compared with the Δ*hly* and Δ*actA* deletion mutant. (**C**) Representative images of the BacLight viability assay of PMNs infected with Δ*hly-Lm* at 2 h 30 min p.i., showing a viable *Lm* stained green with SYTO9 and negative for PI (white arrowhead) and dead/permeabilized *Lm* stained red with PI (empty arrowheads). (**D**) Percentage of viable (SYTO9+/PI–) and non-viable (PI+) WT*-* (dark green and red, from Fig. 2D) and Δ*hly-Lm* (light green and magenta) associated with bovine PMNs. Data are expressed as mean ±SEM from three independent experiments (in which 10 independent FOVs were analyzed per replicate and time point). (**E, F**) Viability of bovine PMNs after infection with WT-*Lm*. Percentage of apoptotic (AV+/PI+ & AV+/PI– [**E**]) and necrotic (AV–/PI+, (**F**)) PMNs. Statistical analyses were performed comparing test conditions with uninfected PMNs. Data were analyzed by Kruskal-Wallis test (**A, B, D–F**) or one-way ANOVA followed by planned comparisons (**C**). * *P* = <0.05; ** = *P* < 0.01; *** = *P* < 0.001; **** = *P* < 0.0001; ns = not significant.

The virulence factors LLO and ActA are crucial for *Lm* intracellular survival, facilitating escape from vacuoles into the cytosol, evasion of xenophagic targeting, and cell-to-cell spread (10, 12, 15). To investigate whether LLO or ActA were involved in *Lm* survival within PMNs, we compared the dynamics of WT-*Lm* and LLO- and ActA-deficient mutants (JF5203-Δ*hly-Lm* and JF5203-Δ*actA-Lm*, referred to as Δ*hly-Lm* and Δ*actA-Lm*) in a gentamicin protection assay. As shown in Fig. 5A, there were no differences in CFU counts between WT-*Lm* and the deletion mutants at any time point. However, CFU counts of Δ*hly-Lm* showed a slight downward trend compared with WT- and Δ*actA-Lm* at later time points. Additionally, no differences were observed between CFUs of serum opsonized and non-opsonized mutants, except for Δ*hly-Lm* at 40 min p.i. (Fig. S2G and H). Likewise, opsonization did not affect intracellular killing of either Δ*hly-Lm* or Δ*actA-Lm* compared with WT-*Lm* (Fig. S2I).

Confocal microscopy revealed similar quantities of non-opsonized and opsonized GFP+ Δ*hly*-, Δ*actA*-, and WT-*Lm* at all time points (Fig. 5B and S2J). Moreover, GFP+ Δ*hly*-, and Δ*actA-Lm* associated with LAMP-1 at rates comparable with GFP+ WT *Lm* (Fig. S2K). Similar to WT-*Lm*, intraneutrophilic bacteria did not produce actin polymerizations.

Because these findings confirmed *Lm’s* incapability to escape from PMN vacuoles and suggested a possible contribution of *hly* to *Lm* intravacuolar survival (Fig. 5A), we focused on Δ*hly-Lm* and performed BacLight assays to examine *hly*’s involvement in bacterial intravacuolar resilience (Fig. 5C). Compared with WT-*Lm*, the percentage of viable SYTO9+/PI– Δ*hly-Lm* was lower at all time points, whereas the percentage of dead PI+ PMN-associated Δ*hly-Lm* was higher (Fig. 5D). These observations suggest that (i) the transition from VBNC to dead bacteria is dynamic and shifts toward bacterial death in the absence of LLO and (ii) *hly* contributes to *Lm* persistence as VBNC bacteria in PMNs, although it is not the sole factor involved. We further investigated *hly* and LLO expression in intraneutrophilic WT-*Lm* through RT-PCR and immunofluorescence. Although *hly* was expressed at 1 h and 5 h p.i. (Fig. S5A), LLO immunolabeling was absent in intraneutrophilic *Lm* at all time points. Conversely, LLO was consistently observed in intracellular *Lm* in a bovine macrophage cell line (BoMacs) infected in parallel (Fig. S5B). This suggests that either intravacuolar LLO is degraded by PMN intravacuolar content or its concentration is too low for detection by immunofluorescence.

Various bacterial pathogens can enhance their survival in PMNs by delaying or promoting PMN cell death (reviewed in reference 46). To determine if phagocytosed *Lm* promote their persistence by influencing PMN cell viability, we analyzed apoptosis and necrosis in bovine PMNs inoculated with viable and dead WT-*Lm* via flow-cytometry (Fig. 5E and F; gating strategy illustrated in Fig. S6). As anticipated, the number of Annexin-V-positive apoptotic cells increased with culture duration in both infected and uninfected PMNs, consistently outnumbering necrotic cells throughout the experiment (Fig. 5E and F). Infection with WT-*Lm*, whether viable or dead, did not significantly increase the apoptotic rate of PMNs compared with the control (Fig. 5E) but did enhance necrosis during early infection (Fig. 5F), likely indicating unspecific non-apoptotic cell death mechanisms (reviewed in reference 47) triggered by phagocytosis. Thus, *Lm* does not actively manipulate bovine PMN apoptosis to facilitate its survival.

### *Lm* can spread from bovine PMNs to BoMacs

To investigate whether persistent intraneutrophilic *Lm* can spread from PMNs to other cells, we seeded *Lm*-infected bovine PMNs onto BoMacs in gentamicin-containing medium to limit infection deriving from extracellular bacteria. In a pilot experiment, CFU counts from PMNs at 24 h 30 min p.i. exposed to high-dose (100 µg/mL) gentamicin for 10 min, a protocol known to rapidly eliminate extracellular *Lm* (40), were similar to those from PMNs treated with 10 µg/mL gentamicin (Fig. S7A). This confirmed that the 10 µg/mL gentamicin concentration was effective in eliminating extracellular bacteria after 24 h 30 min, indicating that the infectious bacteria originated from the intraneutrophilic pool.

**Fig 6:**
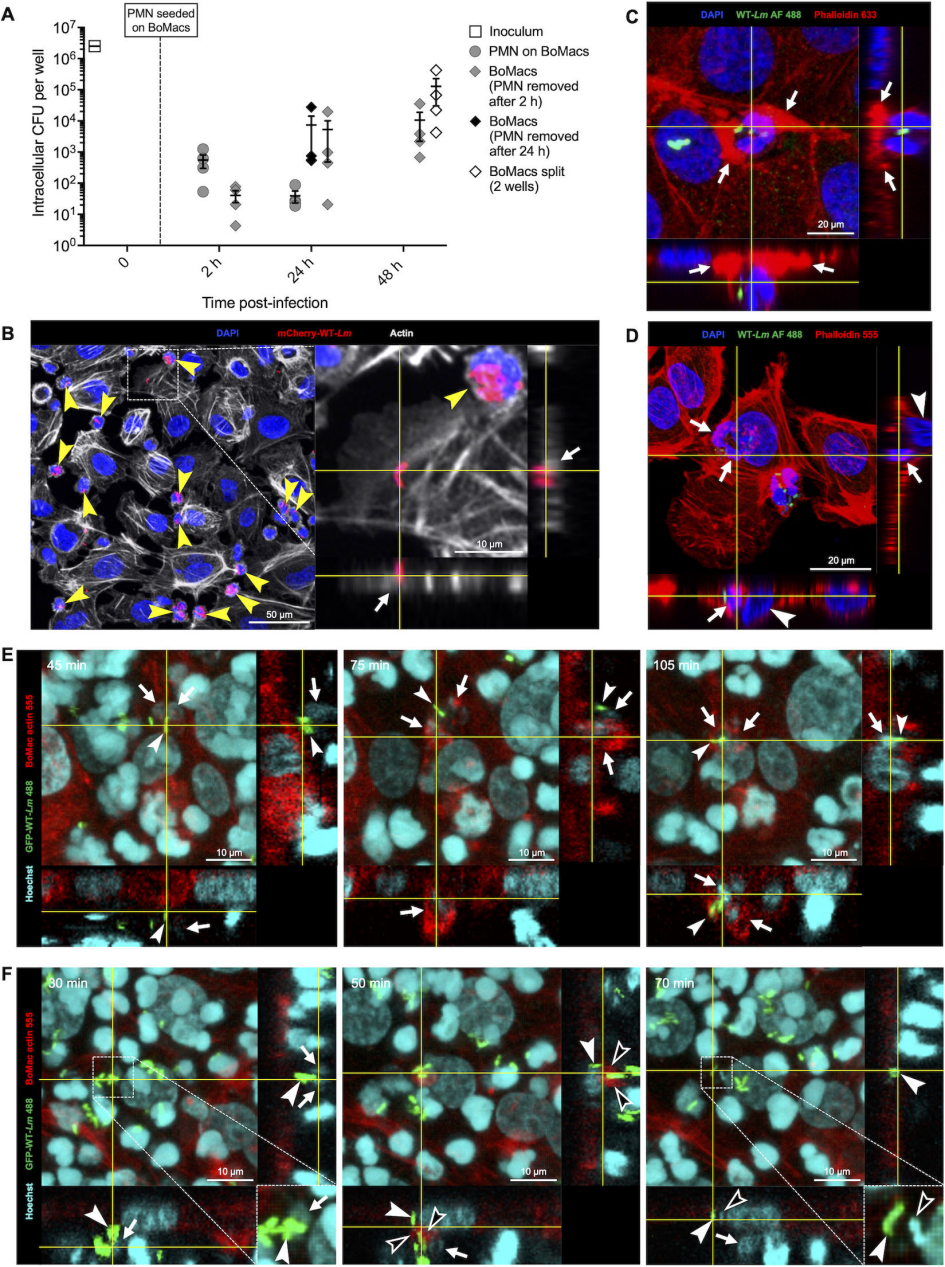
Interaction between *Lm*-infected PMNs and BoMacs results in bacterial transmission. (**A**) PMNs infected with WT-*Lm* for 6 h in the gentamicin assay were seeded on BoMacs. After 2 h or 24 h of co-culture, PMNs were removed by thorough washing and CFU from these PMNs were enumerated (gray circles). CFU from BoMacs co-cultured with PMNs for 2 h prior to PMN removal were enumerated after 2 h of co-culture or after continued culture for 24 h and 48 h (gray diamonds). Additionally, CFU from BoMacs co-cultured with PMNs for 24 h prior to PMN removal (black diamonds) and from BoMacs split into 2 wells at 24 h of co-culture (white diamonds; CFU represent the sum of CFU obtained from each of the two wells) were also enumerated. CFU are normalized to an initial inoculum of 2.5 × 10^6^ CFU. Data are expressed as mean (±SEM) of CFU per well from four independent experiments performed in triplicate. (**B**) PMNs containing mCherry+ WT *Lm* (yellow arrowheads) enter in close contact with BoMacs (larger cells with distinct actin skeleton). Note that the majority of bacteria remain confined to PMNs. Orthogonal views are centered on a diplobacillary mCherry+ *Lm* located on the intracellular side of a BoMac cell membrane (white arrow). Representative MAX intensity projections with orthogonal views, 1 h 30 min of co-culture. (**C**) *Lm*-containing PMN contacted by thick actin structures compatible with BoMac protrusions (white arrows). Actin from both PMN and BoMacs is labeled. Standard deviation (STD) projections with orthogonal views, 2 h of co-culture. (**D**) *Lm*-containing PMN (white arrows) inside a BoMac, resulting in displacement of the BoMac nucleus (white arrowheads). Actin of both PMN and BoMacs is labeled. STD projections with orthogonal views, 2 h of co-culture. (**E**) BoMac internalization of a *Lm*-containing PMN. At 45 min (left panel), a PMN (arrows) associated with GFP-expressing *Lm* (arrowheads) is located extracellularly on top of a BoMac membrane. At 75 min (middle panel) the PMN (arrows) together with its GFP+ *Lm* (arrowhead) is partially engulfed by BoMac actin protrusions (red). At 105 min (right panel), the PMN (arrows) and its associated GFP+-*Lm* (arrowhead) are completely internalized by the BoMac. (**F**) Live-cell imaging showing *Lm* transfer from a PMN to a BoMac without PMN internalization. The left panel (30 min) shows a group of GFP+ *Lm* (arrowheads) associated with a PMN (white arrows, inset; note the shrunken nucleus compared to the adjacent PMN). At 50 min (middle panel), BoMac actin protrusions engulf one of these GFP+ *Lm* (black arrowheads), whereas another GFP+ *Lm* is already internalized (white arrowhead). Note that the PMN nucleus (white arrow) is not internalized. At 70 min (right panel), the PMN-associated bacteria (arrowheads, inset), but not the PMN itself (white arrow), are internalized. One of these *Lm* shows loss of GFP signal (black arrowheads), whereas the other does not (white arrowheads). STD projections with orthogonal views.

After 2 h or 24 h co-incubation, PMNs were removed from BoMacs by thorough washing, and CFUs obtained from BoMacs and from removed PMNs were enumerated. As shown in Fig. 6A, only a small number of bacteria spread from infected PMNs to BoMacs initially, with higher CFU counts in PMNs compared with BoMacs at 2 h of co-culture. Over time, CFU counts decreased in PMNs and increased in BoMacs.

By 24 h, CFU counts were similar between BoMacs co-cultured with PMNs for 24 h and those co-cultured for only 2 h (Fig. 6A), suggesting that most *Lm* are transmitted from PMNs to BoMacs within the first 2 hours. At 48 h, CFU counts were higher in sub-cultured BoMacs wells than in those grown to overconfluence (Fig. 6A), suggesting that cell division might enhance bacterial replication. Altogether, these results indicate that CFU recovered from BoMacs derived from viable intraneutrophilic bacteria and that *Lm* can spread from PMNs to other cells.

To understand the mechanism of *Lm* spread from infected PMNs to BoMacs, we performed confocal microscopy and live-cell imaging. We initially attempted to differentiate cell types using actin stains of different spectra for BoMacs and PMNs (green vs deep red, respectively) infected with mCherry WT-*Lm*. However, actin dye leakage occurred during co-culture. Therefore, cell types (PMNs and BoMacs) were differentiated based on size and morphology (PMNs: small, round to ameboid; BoMacs: larger with oval nuclei and abundant cytoplasm; Fig. 6B through F; Fig. S7B and S8A through E). PMNs were frequently found in close proximity to and in contact with BoMac membranes (Fig. 6B). At all time points, most *Lm* were observed within PMNs, with no actin polymerization detected, confirming their containment in vacuoles. At early time points, BoMacs contained single or few intracellular bacteria, none of which displayed polymerized actin prior to 24 h of co-culture (Fig. 6B; Fig. S7B), indicating early *Lm* transmission and transient containment within BoMacs’ vacuoles. Actin-polymerizing bacteria were first observed at 24 and 48 h, when *Lm* clusters (“*Lm* foci”) spanning multiple adjacent BoMacs were occasionally detected (Fig. S7B and C). These foci formed even when PMNs were removed after only 2 h of co-culture. At 48 h, subcultured BoMacs exhibited more *Lm* clusters compared to non-passaged BoMacs (Fig. S7C), consistent with observations in hepatic cell lines (40), suggesting that cell division might trigger bacterial resuscitation. However, the formation of *Lm* foci appeared to be stochastic, as it was not consistently observed across all experiments (Fig. S7C). Collectively, these data indicate that viable *Lm* transmitted from PMNs to other cells regain the ability to complete their canonical intracytosolic life cycle, which is required for full virulence and efficient spread.

Frequently, PMNs were partially surrounded by actin polymerizations resembling BoMacs protrusions (Fig. 6C), and infected and uninfected PMNs were found inside BoMacs (Fig. 6D). Live-cell imaging of BoMac expressing LifeAct-TagRFP co-cultured with actin-unlabeled PMNs revealed BoMacs exhibiting probing behavior, extending actin protrusions towards PMNs and retracting them without internalizing PMNs (Fig. S8A). Alternatively, BoMacs engulfed PMNs with cup-shaped actin protrusions, eventually internalizing them (Fig. 6D and E; Fig. S8B), indicating that PMNs can be actively taken up by BoMacs. Both *Lm*-infected (Fig. 6E) and uninfected PMNs (Fig. S8B) were internalized, but no *Lm* transmission from internalized PMNs to the BoMacs cytoplasm was observed. *Lm* transmission occurred when BoMacs contacted PMN-associated bacteria with actin protrusions, internalizing bacteria without the PMNs (Fig. 6F). This involved PMNs with shrunken nuclei (Fig. 6F), suggesting loss of cell viability. Similarly, extracellular *Lm* near PMNs with small, round, dense nuclei and dissolving actin (Fig. S8C), as well as groups of *Lm* associated with PMN remnants (Fig. S8D) were observed. Extracellular *Lm* not associated with PMNs were rapidly contacted and internalized by BoMac actin protrusions (Fig. S8E). Interestingly, *Lm* internalized by BoMacs frequently lost their GFP signal (Fig. 6F; Fig. S8E), suggesting that these *Lm* were taken up in BoMacs acidic vacuoles resulting in GFP quenching. A summary and quantification of live-cell imaging events are reported in Table S1 (at https://doi.org/10.48620/84800). The complete live-cell imaging sequences are shown in Video S1.

Taken together, our data suggest that PMNs not only allow for intracellular survival of *Lm* but might also serve as a novel mobile source for re-infection of other cell types, where *Lm* resuscitates from its dormant VBNC state.

## DISCUSSION

PMNs are generally viewed as first responders during acute listeriosis (25, 26, 28, 29), crucial for early bacterial containment and killing (reviewed in reference 26). However, PMNs alone are unlikely to efficiently clear *Lm* without the subsequent contribution of adaptive immune cells (30–33), challenging their perceived efficacy against *Lm*. Increasing evidence suggests that *Lm* can survive in intravacuolar niches within epithelial cells (40, 44) as well as macrophages (43, 48). In particular, persistence of *Lm* within mobile phagocytes may allow bacterial spread and reactivation of infection at new sites under favorable conditions, providing a basis for recurrent and chronic listerial infections (49–51). Therefore, identifying new intracellular niches for *Lm* persistence that may be clinically, pathomechanistically, and epidemiologically relevant is crucial. In light of these findings, the paucity of studies focusing on *Lm* interactions with PMNs at the cellular level (52, 53) is surprising.

Our data show that although bovine PMNs, similar to murine and human PMNs (36, 52), are efficient at reducing the number of culturable bacteria over a short time, they cannot completely eliminate extracellular and intracellular *Lm*. In the absence of gentamicin, bactericidal activity is rapidly saturated, likely due to the continued replication of extracellular bacteria. Even with gentamicin and after bacterial opsonization, PMNs still do not achieve complete elimination of intracellular bacteria.

Interestingly, the majority of surviving *Lm* enter a VBNC state following PMN phagocytosis. VBNC bacteria are dormant cells that retain viability but exhibit low metabolic activity and fail to form colonies on standard growth media (54, 55). *Lm* has been shown to enter the VBNC state in the environment (reviewed in reference 55) and more recently in epithelial cells (40). This state is triggered by exposure to various stressors, and the induction time may vary based on the type of stress. In the aquatic environment, VBNC *Lm* are first detected after 7 days of exposure (56). In contrast, our findings indicate that intraneutrophilic *Lm* begin to switch to VBNC forms within hours of phagocytosis, consistent with other studies where stressors such as non-ionic surfactants or copper induced VBNC forms within minutes to hours (57, 58). Similarly, *Salmonella* persisters have been shown to emerge within minutes after phagocytosis by macrophages (59). The rapid onset of the VBNC state in intraneutrophilic *Lm* correlates with structural differences between VBNC forms. Although intra-aquatic VBNC *Lm* lack a cell wall (56), intraneutrophilic *Lm* retain an intact cell wall, suggesting that the VBNC state may encompass a spectrum of physiological adaptations influenced by the surrounding environment and the stress encountered. Our results provide the first evidence that VBNC bacteria form in PMNs, thereby expanding the range of known intracellular niches for *Lm* persistence. Taken together, our results indicate that due to their limited listericidal activity, PMNs can harbor intracellular persisters of *Lm*. Although the incomplete bactericidal effect of PMNs is not unique to *Lm* and has been reported for other bacteria, such as *Escherichia coli* (60) and *Staphylococcus aureus* (61), the formation of VBNC forms within PMNs for these species has yet to be demonstrated.

Our data provide strong evidence that *Lm* resides in vacuolar compartments of bovine PMNs, similar to those seen in murine PMNs (53). These single-membrane vacuoles are heterogeneous, with some showing features of Spacious Listeria‐containing Phagosomes described in macrophages (SLAPs) (43, 48) and epithelial cells (eSLAPs) (44), and others exhibiting features of so-called LisCVs (40). SLAPs are large, non-acidic, non-degradative phagosomes decorated with LAMP-1 and LC3 (43, 62). In contrast, LisCVs are acidic, partially degradative vacuolar compartments, decorated with LAMP-1 but not LC3b, into which cytosolic *Lm* lacking polymerized actin are caught (40). However, a transient intracytosolic stage of *Lm* was not observed in PMNs at any time point of infection. Our observations indicate that *Lm-*containing vacuoles in PMNs may form through multiple pathways, including conventional phagocytosis (63, 64), in which bacteria-containing phagosomes rapidly fuse with LAMP-1 coated pre-lysosomal compartments, and non-canonical autophagic targeting akin to LC3-associated phagocytosis (LAP) (62). The higher frequency of bacteria in LAMP-1-coated vacuoles suggests that *Lm* predominantly enter the phagolysosomal pathway of PMNs, as previously reported in mice (53). Further characterization of the nature of such vacuoles, as well as identifying the intravacuolar mechanisms promoting either *Lm* persistence or degradation in PMNs, will be crucial to understanding the survival mechanisms of *Lm* in PMNs and may lead to the discovery of potential therapeutics targeting *Lm* persisters.

To further understand mechanisms of intraneutrophilic persistence, we investigated the modulation of PMN death described in a variety of bacterial pathogens (46, 65) and the impact of bacterial virulence factors. Our data indicate that *Lm* does not actively influence bovine PMN apoptosis and necrosis. The most important *Lm* virulence factor for vacuolar escape is LLO, and insufficient LLO causes intravacuolar containment of *Lm*. Immunofluorescence did not reveal any bacterial LLO protein expression within PMNs despite transcript detection of the LLO encoding gene *hly*, which is in line with the observed degradation of LLO by MMP-8 from human PMNs (52). Interestingly, deletion of *hly* decreased the ratio of intravacuolar VBNC to dead bacteria in PMNs, indicating that LLO is involved in intravacuolar survival as VBNC bacteria. Therefore, it is tempting to speculate that very low intravacuolar LLO levels, not detectable by immunofluorescence, may contribute to *Lm* intravacuolar persistence but are not sufficient for vacuolar escape (43). Whether *Lm* is capable of adopting further strategies for intravacuolar survival in PMN similar to those observed in other bacterial species (reviewed in references 46, 66) requires further investigations.

The discovery of intraneutrophilic VBNC persisters of *Lm* led us to explore whether PMNs can transmit *Lm* to other cells. Our findings reveal that intraneutrophilic *Lm* can spread to co-cultured cells, albeit at low rates, where they may resuscitate, regaining the ability to replicate in the cytosol and spread further. *Lm* transmission events were linked either to the uptake of bacteria from PMNs that displayed loss of cellular integrity or directly from extracellular *Lm* released upon PMN death. In the latter case, bacteria appear capable of surviving briefly in the gentamicin-containing medium, sufficient for uptake by neighboring cells. Additionally, we observed internalization of uninfected or *Lm*-containing PMNs by co-cultured cells. Although not observed, we cannot exclude *Lm* transmission events from internalized PMNs. Although the clinical relevance of these transmission mechanisms requires validation through *in vivo* studies, phagocytosis of apoptotic PMNs by macrophages (efferocytosis) has been observed in patients with listerial meningitis (67). Following efferocytosis, persistent intraneutrophilic *Lm* may survive in macrophages, akin to *Yersinia pestis* (68). Furthermore, extracellular VBNC *Lm* released from dead PMNs may directly infect macrophages and replicate, as shown for *Legionella pneumophila* (69), potentially gaining access to further mobile cellular niches. The formation of VBNC *Lm* in mobile PMNs represents a potentially sophisticated survival strategy providing the significant advantage of persisting and disseminating in the host while remaining undetectable to the immune system and shielded from antibiotics (70, 71). Infected PMNs may spread persistent *Lm* locally upon their death or act as Trojan horses, disseminating *Lm* to distant sites, as proposed for *Staphylococcus aureus* (reviewed in reference 72). Reverse PMN migration (73–75) could facilitate the movement of *Lm*-infected PMNs from infection sites into the bloodstream or lymphatics, offering a potential source for sporadic and recurrent listeriosis when *Lm* escapes from PMNs. A similar mechanism has been previously speculated for epithelial cells (40). Moreover, VBNC *Lm* may remain clinically undetected due to failure of culture from patient samples (76), potentially explaining the low success rate of *Lm* isolation from cerebrospinal fluid in humans and ruminants with listerial rhombencephalitis (77–80).

In conclusion, our findings reveal a previously unrecognized dual role of PMNs in listeriosis, acting as a double-edged sword with both protector and facilitator properties. Although PMNs exhibit strong listericidal activity, significantly reducing bacterial load, they paradoxically allow a small population of *Lm* to survive intracellularly, persist as VBNC forms, spread, and constitute a new infective population. This positions PMNs as a novel niche for *Lm* survival and potential dissemination, creating diagnostic and therapeutic challenges due to the enigmatic VBNC state. Further research is essential to understand the clinical implications of intraneutrophilic VBNC *Lm* and their contribution to listeriosis pathophysiology. Addressing these knowledge gaps may prove crucial for identifying targets for novel therapeutic interventions and improving diagnostic strategies to better manage this complex and persistent pathogen.

## MATERIALS AND METHODS

### Bovine and human PMN isolation

Bovine blood was collected by jugular venipuncture from cows at the Clinic for Ruminants of the Vetsuisse Faculty, University of Bern (Switzerland). PMN were isolated by Ficoll-Paque PREMIUM 1.084 (GE Healthcare, Chicago, IL) density gradient centrifugation, followed by erythrocyte lysis in hypotonic lysis buffer (8.29 g/L NH_4_Cl, 1 g/L NaHCO_3_, pH 7.4), as previously described (27).

Human blood was obtained from the Interregional Blood Transfusion Company of the Swiss Red Cross, Bern, Switzerland. Human PMNs were isolated through Ficoll-Hypaque (1.077 g/mL) density centrifugation, followed by erythrocyte lysis in sterile water, as previously described (81).

The purified PMNs were resuspended in medium consisting of Dulbecco’s modified Eagle’s medium (DMEM; Ref: 31600-083, Gibco, Life Technologies; 10 g/L) containing NaHCO_3_ (2 g/L), adjusted to pH 6.8, and supplemented with 10% heat-inactivated fetal calf serum (FCS; Bioswisstec, Schaffhausen, Switzerland) and 1% L-glutamine 0.2 M (Merck Millipore, Darmstadt, Germany). PMN number and viability were assessed using a CASY Cell Counter (OLS OMNI Life Science, Bremen, Germany) (PMN purity averaged approximately 90%), and PMNs were incubated for 30 min at 37°C on a shaking platform to allow recovery before use.

### BoMacs cell culture

The immortalized bovine macrophage cell line “BoMac” (82) was grown in DMEM supplemented with 10% FCS, 100 U/mL penicillin, and 10 µg/mL streptomycin (Life Technologies). Cells were seeded in 24-well plates and grown to confluence overnight, then washed three times with PBS, and incubated with fresh medium without antibiotics before infection with *Lm* or co-culture with *Lm*-infected PMN. To directly visualize BoMac actin during live-cell imaging, we generated BoMacs constitutively expressing Lifeact-TagRFP. Briefly, cultured BoMacs were transfected with Lifeact-TagRFP (Ibidi GmBH) conjugated to the pRRL expression plasmid using the TransIT-LT1 Transfection Reagent (Mirus, Madison, WI) according to the manufacturer’s instructions. Transfected cells were selected via a limiting dilution.

### Bacterial strains and preparation for infection assays

The *Lm* strain JF5203 (lineage I, clonal complex 1, sequence type 1, https://www.ncbi.nlm.nih.gov/nuccore/NZ_LT985474.1; referred to as WT-*Lm* throughout the paper) isolated from a case of bovine rhombencephalitis and previously generated isogenic *hly* and *actA* deletion mutants (JF5203-Δ*hly*, JF5203-Δ*actA*; referred to as Δ*hly-Lm* and Δ*actA-Lm*, respectively) (27, 28) were used. Green fluorescent protein (GFP) expressing variants of the parental strain (GFP JF5203-WT-*Lm*, hereafter referred to as GFP WT-*Lm*) and of isogenic deletion mutants (GFP JF5203-Δ*hly-Lm* and GFP JF5203-Δ*actA-Lm*, referred to as GFP Δ*hly-Lm* and GFP Δ*actA-Lm*, respectively) were generated by transformation with the pPL2-Phyper-GFP plasmid as previously described (28, 83). A mCherry expressing a variant of the parental strain (mCherry JF5203-WT-*Lm*, referred to as mCherry WT-*Lm*) was generated as previously described by transformation with a pPL2-mCherry plasmid developed by the group of John-Demian Sauer (Department of Medical Microbiology and Immunology, University of Wisconsin-Madison, USA) (84) and expressed in *E. coli* (*E. coli* SM10-pPL2-mCherry, kindly provided by Hélène Bierne, Université Paris-Saclay, INRAE, France). Depending on the experiment, non-fluorescent, GFP or mCherry strains were used for microscopy. Single *Lm* colonies were inoculated and grown overnight in brain-heart infusion (BHI) broth, diluted, and in a subset of experiments opsonized with fresh bovine serum as previously described (27). The concentration of bacterial inoculum was confirmed for each experiment by plating an aliquot onto BHI-agar plates and enumerating colony-forming units (CFU) after overnight incubation at 37°C. All experiments were performed at a multiplicity of infection (MOI) of 5:1, except for selected assays where an MOI of 0.1:1 was used.

### Killing assay

Bovine PMNs were resuspended in a medium, and triplicates of 10^5^ PMNs were seeded together with an inoculum of 5 × 10^5^ CFU (MOI: 5) or 10^4^ CFU (MOI: 0.1) of WT-*Lm* per well in 96-well flat-bottomed plates (Corning, Vitaris, Baar, Switzerland). To assess bacterial killing, wells containing PMN and WT-*Lm* were compared with control wells containing 5 × 10^5^ CFU (MOI: 5) or 10^4^ CFU (MOI: 0.1) of WT-*Lm* without PMNs in each experiment. Plates were centrifuged at 300 × *g* for 5 min at RT to synchronize phagocytosis and then incubated at 37°C in 5% CO_2_. CFU were measured at 10 min, 30 min, 1 h, 2 h, 5 h, and 24 h post-infection (p.i.) by plating of serial dilutions (1:1–1:10^6^) on BHI agar after thorough resuspension of PMNs and bacteria. CFU were counted after 24 h of incubation at 37°C, and the raw CFU counts obtained were normalized to the respective inoculum to allow comparison between experiments.

### Gentamicin protection assay

Bovine or human PMNs and bacteria (either mutant or the parental strain) were diluted and plated as described above. Phagocytosis was allowed to occur for 30 min, after which gentamicin (Sigma-Aldrich) was added to each well to a final concentration of 10 µg/mL to kill extracellular bacteria. This concentration of gentamicin was sufficient to kill ~90% of the bacteria (relative to the initial inoculum) within 10 min of addition (Fig. 1E). The post-infection time points evaluated in this assay for bovine PMNs were: 40 min (10 min after gentamicin addition), 1 h, 1 h 30 min, 2 h 30 min, 5 h 30 min, and 24 h 30 min p.i. For human PMN, 2 h 30 min and 5 h 30 min were evaluated. At each time point, 100 µL containing resuspended bacteria and PMNs were collected from each well and transferred to a new 96-well plate, centrifuged at 1,000 × *g* for 2 min to allow PMNs to adhere to the bottom of the well, then the gentamicin-containing medium with residual extracellular bacteria was discarded. PMNs were washed with 100 µL of gentamicin-free medium, resuspended, and centrifuged again, after which the medium was removed and PMNs were lysed by adding 100 µL of sterile water to each well. Between each step, the plates were examined microscopically to confirm that PMNs had not been washed away. PMN lysis was assessed microscopically prior to serial dilutions, and plating and CFU enumeration were performed as described above.

### Immunofluorescence assays

To microscopically evaluate the interaction between bovine PMNs and intracellular *Lm*, a gentamicin protection assay with bacteria and PMNs seeded on glass coverslips (12 mm diameter, Carl Roth) was performed as described above, with the following modifications, and 250 µL of bacterial suspension (2.5 × 10^6^ CFU/well) was co-cultured with 250 µL of PMN (5 × 10^5^ PMNs/well) on poly-L-lysine-coated coverslips in a 24-well plate (Corning, Vitaris, Baar, Switzerland). After phagocytosis, synchronization with centrifugation at 300 × *g* for 5 min at RT and gentamicin supplementation at 30 min p.i., all or selected of the following time points post-infection were analyzed depending on the assay performed: 1 h p.i. (30 min after gentamicin addition), 1 h 30 min p.i., 2 h 30 min p.i., 5 h 30 min p.i., and 24 h 30 min p.i. For each time point, the medium was removed from each well, PMNs were air dried for 5 min and fixed in 4% paraformaldehyde (PFA; Sigma-Aldrich) overnight, and then processed for immunofluorescence. For assays to assess LLO expression, bovine PMNs or BoMacs grown to confluence on glass coverslips were infected with *Lm* (WT-*Lm* or Δ*hly-Lm*) at an MOI of 5, processed and fixed at 2 h 30 min, 5 h 30 min, and 24 h 30 min p.i. as indicated previously.

Fixed cells on coverslips were washed three times with PBS containing 0.5% Tween (PBS-T) and permeabilized in PBS containing 0.2% Triton X‐100 for 30 min at RT. Nonspecific antibody binding was prevented by incubating cells in PBS‐T containing 10% normal goat serum (NGS; Dako, Baar, Switzerland) for 30 min (LAMP-1, LLO) or 2 h (LC3b) at RT before incubation with rabbit anti-LAMP-1 antibody (ab24170, Abcam, 1:100), rabbit-anti-LC3b (PA1-46286, Invitrogen, 1:100), rabbit anti-LLO (Abcam; 1:100), or rabbit anti-Listeria polyclonal antibody (1:200, Difco Laboratories, Detroit MI, USA; used in assays in which GFP- or mCherry-expressing *Lm* were not used) in PBS-T containing 10% NGS for 1 h at RT. Coverslips were washed three times with PBS-T and incubated with Alexa Fluor (AF) 488 or 555‐conjugated goat anti‐rabbit IgG secondary antibody (Life Technologies, 1:500), Alexa Fluor 633-conjugated phalloidin (Invitrogen, 1:500), and DAPI (Invitrogen, 1:10,000) for 1 h at RT in the dark. Cells were washed three times in PBS-T, then coverslips were rinsed in distilled water, dried, and mounted with Glycergel mounting medium (Dako, Glostrup, Denmark) onto glass microscope slides (Menzel‐Gläser). Cells were imaged using an Olympus Fluoview FV3000 confocal laser scanning microscope (Olympus, Tokyo, Japan), equipped with the following laser channels: 405, 488, 566, and 647 nm. For each coverslip in which *Lm* were enumerated, 10 or 20 random, non-overlapping fields of view (FOV) were imaged at a 60× magnification using an optimization zoom of 2.31. Images were analyzed using open-source software Fiji, and the number of PMN and *Lm* and their association with each marker were enumerated using the Fiji cell counter plugin (85). GFP+ bacteria were identified by their GFP expression and DAPI staining, whereas GFP– bacteria were identified by their DAPI staining and lack of GFP expression. The total number of PMNs and bacteria analyzed from this and the microscopy assays described below is shown in Table S2 (at https://doi.org/10.48620/84800). To allow for an approximate comparison between the number of microscopically enumerated bacteria and CFU counts over time, the sum of *Lm* counted in 10 or 20 FOVs analyzed for each condition and time point was divided by the total number of PMNs from those same FOVs. This provided the average number of *Lm* per PMN. The value was then normalized to (i.e., multiplied by) 10^5^ to approximate the number of PMNs seeded per well for CFU counting. This normalization procedure was also applied to all subsequent microscopy assays. For assays in which LLO was assessed, the *Lm* count was not enumerated.

### Bacterial viability assays

Viability of intraneutrophilic *Lm* was assessed using a LIVE/DEAD BacLight Bacterial Viability Kit (L7012, Life Technologies) according to a previously described protocol (41) with minor modifications. Briefly, bovine PMNs and *Lm* (WT-*Lm* or Δ*hly-Lm*) were co-incubated on glass coverslips adopting procedures and time points described above for the immunofluorescence assays. After incubation, PMNs were washed twice with 0.1 M 3-(N-morpholino) propanesulfonic acid (MOPS), pH 7.2, with 1 mM MgCl_2_ (MOPS/MgCl_2_) and incubated with Live/Dead Staining Solution, composed of 1.6 µM SYTO9, 20 µM propidium iodide (PI), and 0.1% saponin (final concentrations) in MOPS/MgCl_2_ for 15 min at RT in the dark. Cells were then rinsed three times in MOPS/MgCl_2_, and coverslips were inverted onto glass slides and sealed with clear nail polish for 2 min before confocal imaging for a maximum of 30 min each. Ten random, non-overlapping FOVs were imaged per coverslip at 60× magnification with an optimization zoom of 2.31. The acquired images were then processed with Fiji as described above. Staining efficacy was confirmed by the 100% PI-positivity of WT-*Lm* killed in 70% isopropanol for 10 min compared with untreated WT-*Lm*, in which only individual bacteria showed PI-positivity (not shown), as previously described (41). To verify that live *Lm* were intracellular, differential staining for extra- and intra-cellular *Lm* was performed concurrently with the BacLight assay at 2 h 30 min, 5 h 30 min, and 24 h 30 min p.i. Bovine or human PMNs were rinsed three times in PBS and then incubated for 30 min in PBS containing rabbit anti-Listeria antibody (1:200) supplemented with 10 µg/mL gentamicin. Cells were washed three times in PBS and incubated in PBS containing Alexa Fluor 647‐conjugated goat anti‐rabbit IgG secondary antibody (Life Technologies, 1:500) and 10 µg/mL gentamicin for 30 min, then rinsed three times in MOPS/MgCl_2_ and processed for the viability staining as described above. Z-stack images of 5 independent FOVs per coverslip were acquired and processed as described above for the viability staining.

### Digitonin permeabilization assay

To confirm that intraneutrophilic *Lm* were located in the vacuoles and not in the PMN cytosol, a differential permeabilization assay with digitonin was performed with slight modifications to a previously described protocol (42). In this assay, the treatment of cells with digitonin after bacterial phagocytosis allows selective permeabilization of the plasma membrane while leaving vacuolar membranes intact. It is therefore possible to immunolabel extracellular and intracytosolic (i.e., extravacuolar) bacteria after permeabilization, whereas intravacuolar bacteria remain unstained (42). Briefly, bovine PMNs were incubated with GFP WT-*Lm* for 2 h 30 min and 24 h 30 min as described above for the immunofluorescence assay. At each time point, PMNs were washed three times with KHM buffer (110 mM potassium acetate, 20 mM HEPES, 2 mM MgCl_2_, pH 7.3) and permeabilized for 1 min with 50 µg/mL digitonin (Cat# D5628, Sigma-Aldrich) in KHM buffer. During this step, unpermeabilized PMNs were incubated for 1 min in KHM buffer without digitonin. PMNs were immediately washed three times with KHM buffer and then incubated with rabbit anti-*Listeria* antibody (1:200) in KHM supplemented with 3% bovine serum albumin (BSA, Cat# A2153, Sigma-Aldrich) for 15 min at 37°C in 5% CO_2_. PMNs were washed three times with PBS, fixed with 4% PFA for 10 min, and washed three times with PBS and consecutively three times with PBS supplemented with 3% BSA and 0.1% saponin. PMNs were then incubated in PBS with 3% BSA and 0.1% saponin containing secondary antibody (Alexa Fluor 555 goat-anti-rabbit IgG, 1:500), DAPI (1:10000), and Alexa Fluor 633-conjugated phalloidin (1:500) for 1 h at RT. The coverslips were then washed three times with PBS and mounted as described for the immunofluorescence assay. Z-stacks of 10 random FOV were taken and analyzed with Fiji as described above. Permeabilization efficacy was tested in each assay by staining PMNs with Hoechst 33342 (Invitrogen, Cat. No. H21492, 1:500) and PI (100 nM) for 15 min at 37°C in 5% CO_2_ and by microscopically evaluating PI-positive nuclear staining of permeabilized PMNs and PI-negative nuclear staining in unpermeabilized PMNs.

### LysoTracker assay

The pH of *Lm*-containing vacuoles was assessed using LysoTracker Red DND-99 (#L-7528, Invitrogen) following the manufacturer’s instructions. Briefly, bovine PMNs were incubated for 2 h 30 min and 24 h 30 min as described for the immunofluorescence assay. At each time point, the medium was removed from each well, and PMNs were incubated in medium supplemented with 50 nM LysoTracker Red DND-99 for 30 min at 37°C in 5% CO_2_, followed by three washes with PBS and fixation with PFA for 15 min at RT. PMNs were then stained for LAMP-1 as described above using Alexa Fluor 647‐conjugated goat anti‐rabbit IgG as the secondary antibody. Ten independent FOVs were imaged per coverslip and analyzed as described above.

### Transmission electron microscopy (TEM)

To further characterize the nature of *Lm*-containing vacuoles, bovine PMNs were seeded in 6-well plates (Corning, Vitaris, Baar, Switzerland) at a density of 3 × 10^6^ PMNs per well and co-incubated with 1.5 × 10^7^ WT-*Lm* per well (MOI 5) for 2 h 30 min and 24 h 30 min in the presence of gentamicin, as described above. At each time point, PMNs were processed for TEM and imaged as previously described (86). For each experiment and at each time point, >20 PMNs were imaged. Images of naturally occurring cases of listeriosis were either obtained after sample processing using the same protocol or were obtained using alternative, previously published procedures (28).

Analysis of *hly* gene expression by reverse-transcription (RT)-PCR *hly* gene mRNA expression of intraneutrophilic *Lm*, as well as that of the control genes *16* s and *gyrA*, was assessed by RT-PCR at 1 h p.i. and 5 h p.i. with a total of 1.8 × 10^7^ PMN with 9 × 10^7^ WT-*Lm* (MOI 5) seeded in a 6-well plate (Corning, Vitaris, Baar, Switzerland) in a gentamicin assay as described above. Processing of *Lm*-infected cells, RNA extraction, and reverse transcription using previously described fw and rv primers for 16s, *gyrA,* and *hly* were performed exactly as described previously (27, 87).

### PMN viability and apoptosis assays

To assess how *Lm* infection modulates PMNs death and apoptosis, PMNs were inoculated with WT-*Lm* or WT-*Lm* fixed overnight in 4% paraformaldehyde (PFA-fixed WT-*Lm*) as described above for the immunofluorescence assays. Non-viability of PFA-fixed WT-*Lm* was assessed by lack of colony growth after plating and overnight incubation. Bovine PMNs incubated in medium alone or with 1 µM staurosporine (Cat# S5921, Sigma-Aldrich) were used as negative or positive controls, respectively. PMNs were incubated under the above conditions for the following times: 2 h 30 min, 5 h 30 min, and 24 h 30 min. At each time point, PMN viability was assessed using an Annexin-V-FITC/PI Apoptosis Detection Kit (ab14085; Abcam, USA) and stained according to the manufacturer’s instructions. PMNs were then harvested and measured using an Attune NxT Flow Cytometer (Thermofisher Scientific, Switzerland). Data were analyzed using FlowJoTM software (Tree Star, Ashland, OR, USA). As shown in Fig. S6, PMN were initially gated on an FSC-A vs SSC-A plot to exclude contaminants, then PMN were gated on an FSC-A vs FSC-H plot in order to include only single PMNs in the analysis (as previously described [27]). Single PMNs were then further gated for annexin-V (AV) and PI positivity using unstained PMNs samples (AV–/PI–) as controls. PMNs were grouped together as apoptotic if they were AV+/PI– PMNs (early apoptotic) or AV+/PI+ PMNs (late-apoptotic), whereas AV–/PI+PMNs were regarded as necrotic.

### Co-culture of *Lm*-infected PMNs with BoMacs

PMNs infected for 6 h with WT-*Lm* in a gentamicin protection assay (as described previously) were resuspended in medium through gentle pipetting and were seeded together with their gentamicin-containing medium on top of confluent BoMacs in 24-well plates. Plates containing BoMacs and PMNs were centrifuged as described above to synchronize infection and cultured for a further 2 h and 24 h. At each time point, the BoMac layer was thoroughly washed three times with PBS to remove any remaining PMN. After microscopic control of PMN detachment, PMNs collected from BoMacs and BoMacs were lysed in 0.5% Triton-X100 (Sigma-Aldrich) in ice-cold ddH_2_O, serially diluted and plated on BHI agar plates for CFU counting. In this assay, the following conditions were assessed: (i) CFU of PMNs co-cultured with BoMacs for 2 h and 24 h; (ii) CFU of BoMacs co-cultured with PMNs for 2 h prior to PMN removal, for which BoMacs were lysed and plated at 2 h, 24 h, or 48 h following their co-culture with PMNs; (iii) CFU of BoMacs co-cultured with PMNs for 24 h prior to PMN removal; and (iv) CFU of BoMacs co-cultured with PMNs for 2 h prior to PMN removal, split into 2 wells at 24 h and cultured until 48 h before being lysed and plated (for this condition, the sum of CFU from both wells is shown in Fig. 6A).

To microscopically assess the interaction of BoMacs with infected PMNs upon co-culture, BoMacs were grown on glass coverslips as described above and stained with Cellmask Green Actin Tracking Stain (1:1,000; Invitrogen, Thermo Fisher Scientific Inc., Waltham, MA, USA) for 30 min, according to the manufacturer’s instructions. PMNs were infected with mCherry-WT-*Lm* for 6 h as described above, followed by staining with CelLmask Deep Red Tracking Stain (1:1,000; Invitrogen, Thermo Fisher Scientific Inc., Waltham, MA, USA) for 30 min, according to the manufacturer’s instructions. PMNs were harvested, seeded on BoMacs, and co-cultured in gentamicin-containing medium for the following time points: 30 min, 1 h, 1 h 30 min, 2 h, 2 h 30 min, 3 h, 3 h 30 min, 4 h, 15 h, and 24 h. At each time point, the cells were fixed and stained with DAPI, and coverslips were mounted and visualized as previously described. Alternatively, PMNs were infected with WT-*Lm* for 6 h, co-cultured with BoMacs in gentamicin-containing medium for 2 h (after which, in selected experiments, BoMacs were cultured until 24 h when they were split into two wells or not, and 48 h), 24 h and 48 h. At each time point, BoMacs were washed to remove non-adherent PMNs, and the cells were fixed and stained with anti-*Lm* antibody, Phalloidin (555 or 633), and DAPI as described above. Z-stacks of representative FOVs from each time point were acquired and analyzed with Fiji.

For live-cell imaging, PMNs were infected with GFP WT-*Lm* for 6 h as described above, after which they were collected and seeded onto Lifeact-TagRFP-expressing BoMacs grown to confluence in µ-Plate 96-well plates (Ibidi) in gentamicin-containing medium. PMNs and BoMacs nuclei were stained with Hoechst as described above. Plates were then incubated at 37 ° C and 5% CO_2_ in an Olympus Fluoview FV3000 confocal laser scanning microscope incubation chamber and imaged at 60× magnification utilizing a resonant scanner. Z-stacks of FOVs of interest were captured every 10 min (nine consecutive sequences/FOV) or 15 min (six consecutive sequences/FOV) starting at 30 min of co-culture or at 4 h of co-culture (for the latter, imaging was initiated immediately after PMNs were removed through washings at 4 h) and were analyzed with Fiji. The number of videos obtained is indicated in Table S1 (at https://doi.org/10.48620/84800).

### Data analysis and graphic representation

The raw data used to produce the graphs are shown in Table S3 (at https://doi.org/10.48620/84800). Statistical analyses were performed using the NCSS 10 Statistical Software (Kaysville, UT, USA, ncss.com/software/ncss.). The normal distribution of the data was assessed using the Shapiro-Wilk test, and differences between groups were analyzed using either the Kruskal-Wallis test or one-way ANOVA followed by planned comparisons. For Kruskal-Wallis and ANOVA analysis, Dunn’s test and Bonferroni test were used, respectively, to correct *P*-values for multiple comparisons. Differences were considered statistically significant at *P* < 0.05, and *P*-values are indicated by asterisks in the figures: *, *P* < 0.05; **, *P* < 0.01; ***, *P* < 0.001; ****, *P* < 0.0001.

Graphs were generated using GraphPad Prism v.9 (GraphPad Software, CA, USA).

## SUPPLEMENTAL MATERIAL

Fig. S1 mbio.02700-24-s0001.tiff Bovine PMNs fail to efficiently sterilize extracellular *Lm*.

Fig. S2 mbio.02700-24-s0002.tiff Serum opsonization has a moderate to no effect on PMN listericidal efficacy and on *Lm* fitness.

Fig. S3 mbio.02700-24-s0003.tiff *Lm* persist as VBNC forms in human PMNs.

Fig. S4 mbio.02700-24-s0004.tiff *Lm* localization in bovine PMN vacuoles is confirmed by selective digitonin permeabilization.

Fig. S5 mbio.02700-24-s0005.tiff The *hly* transcript is expressed by intraneutrophilic *Lm* without concomitant LLO protein detection by immunofluorescence.

Fig. S6 mbio.02700-24-s0006.tiff PMN gating strategy and analysis of bovine PMNs viability after infection with WT-*Lm*.

Fig. S7 mbio.02700-24-s0007.tiff *Lm* transmitted from PMNs to BoMacs can establish infectious foci and regain the ability to spread from cell to cell.

Fig. S8 mbio.02700-24-s0008.tiff Representative images of BoMac interactions with *Lm*-infected PMNs.

Legends mbio.02700-24-s0009.docx Legends for supplemental figures and video.

Video S1 mbio.02700-24-s0010.mp4 Collated movies of live-cell imaging depicting the whole recorded sequences illustrated in the figures.

ASM does not own the copyrights to Supplemental Material that may be linked to, or accessed through, an article. The authors have granted ASM a non-exclusive, world-wide license to publish the Supplemental Material files. Please contact the corresponding author directly for reuse.
