# Supplementary figures and images for: Intravacuolar persistence in neutrophils facilitates Listeria monocytogenes spread to co-cultured cells
Source: mBio. 2025 Mar 11;16(4):e02700-24. doi: 10.1128/mbio.02700-24 (PMC11980584; doi:10.1128/mbio.02700-24)

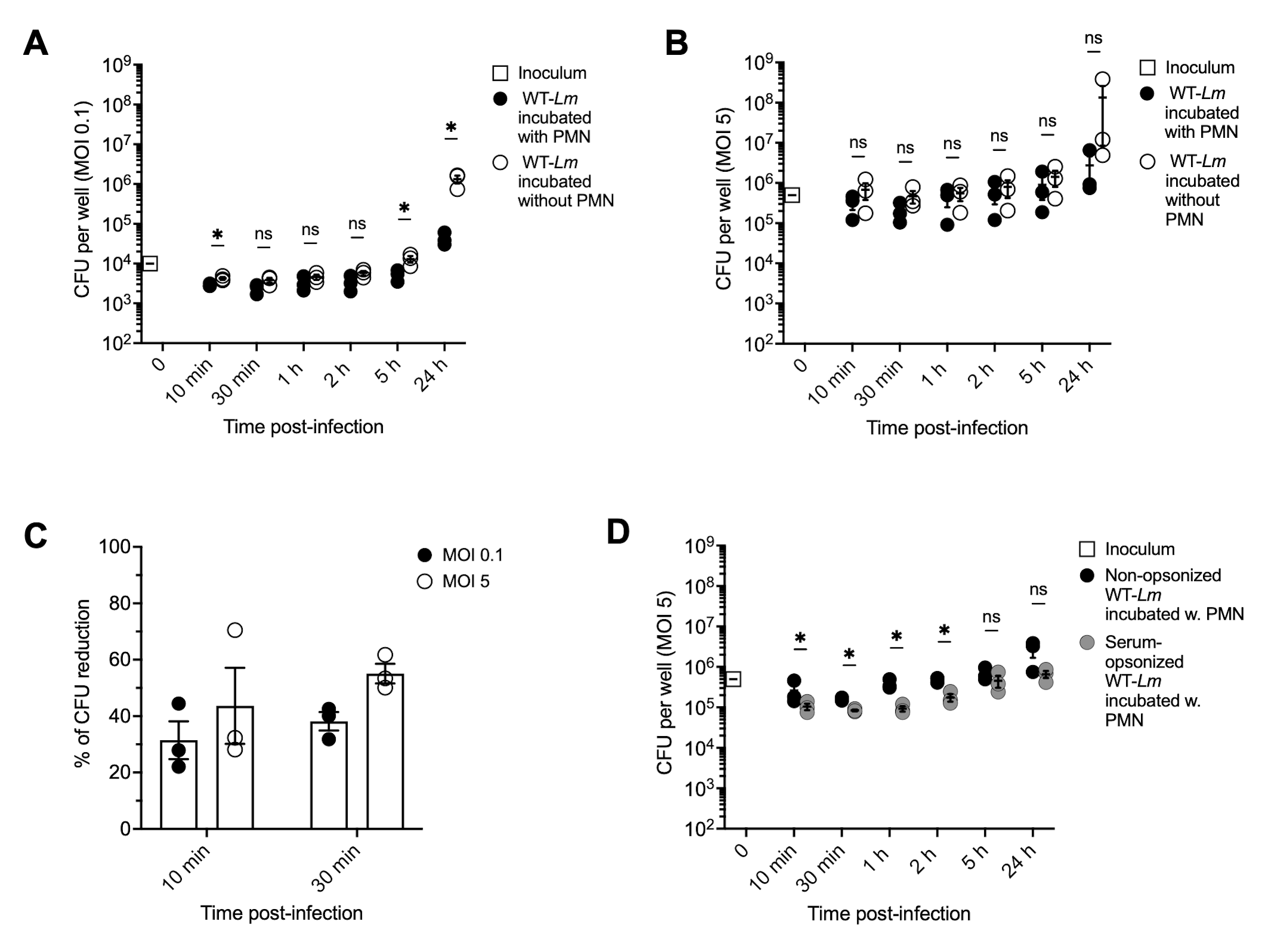

Supplement: Fig. S1 — Bovine PMNs fail to efficiently sterilize extracellular Lm. [file mbio.02700-24-s0001.tiff]

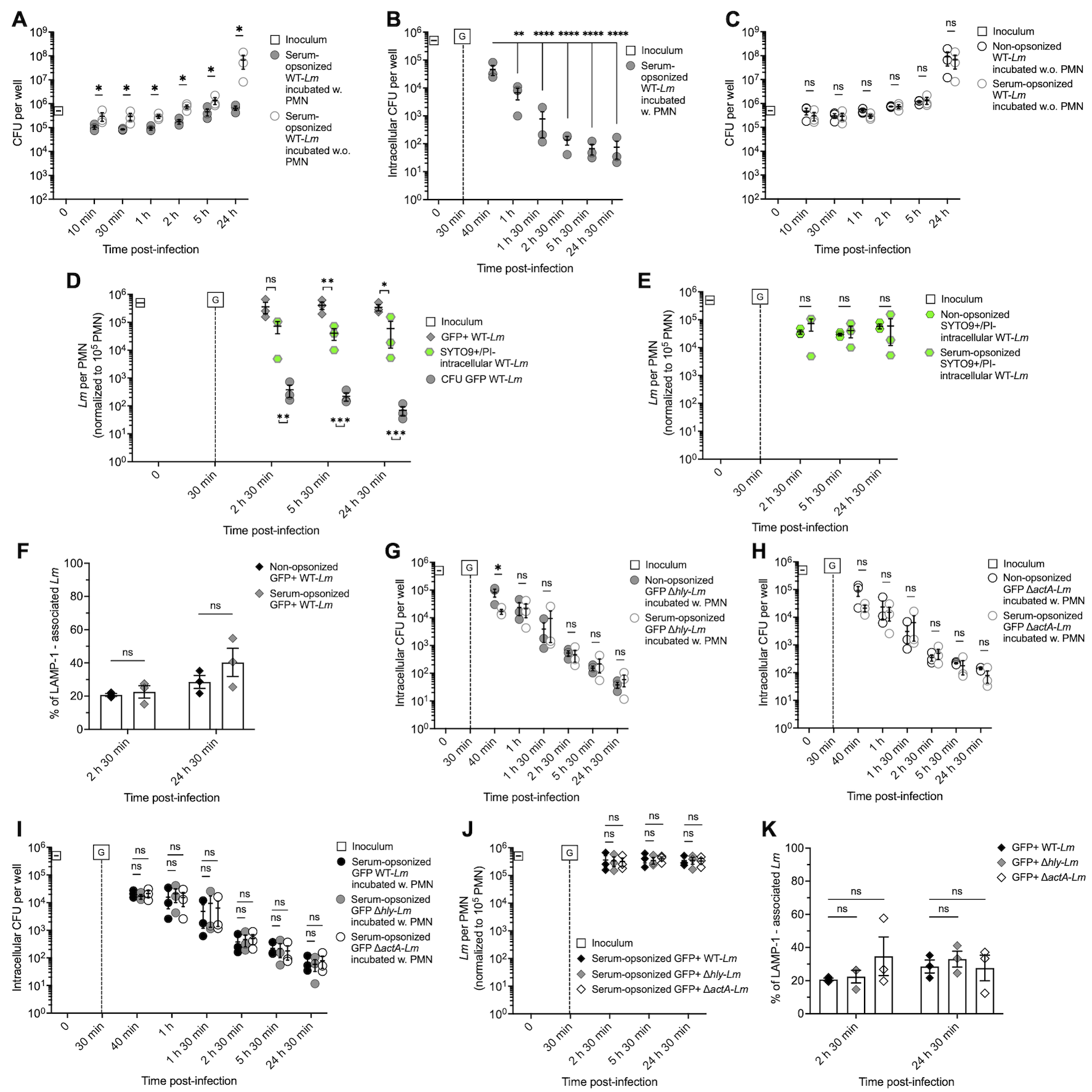

Supplement: Fig. S2 — Serum opsonization has a moderate to no effect on PMN listericidal efficacy and on Lm fitness. [file mbio.02700-24-s0002.tiff]

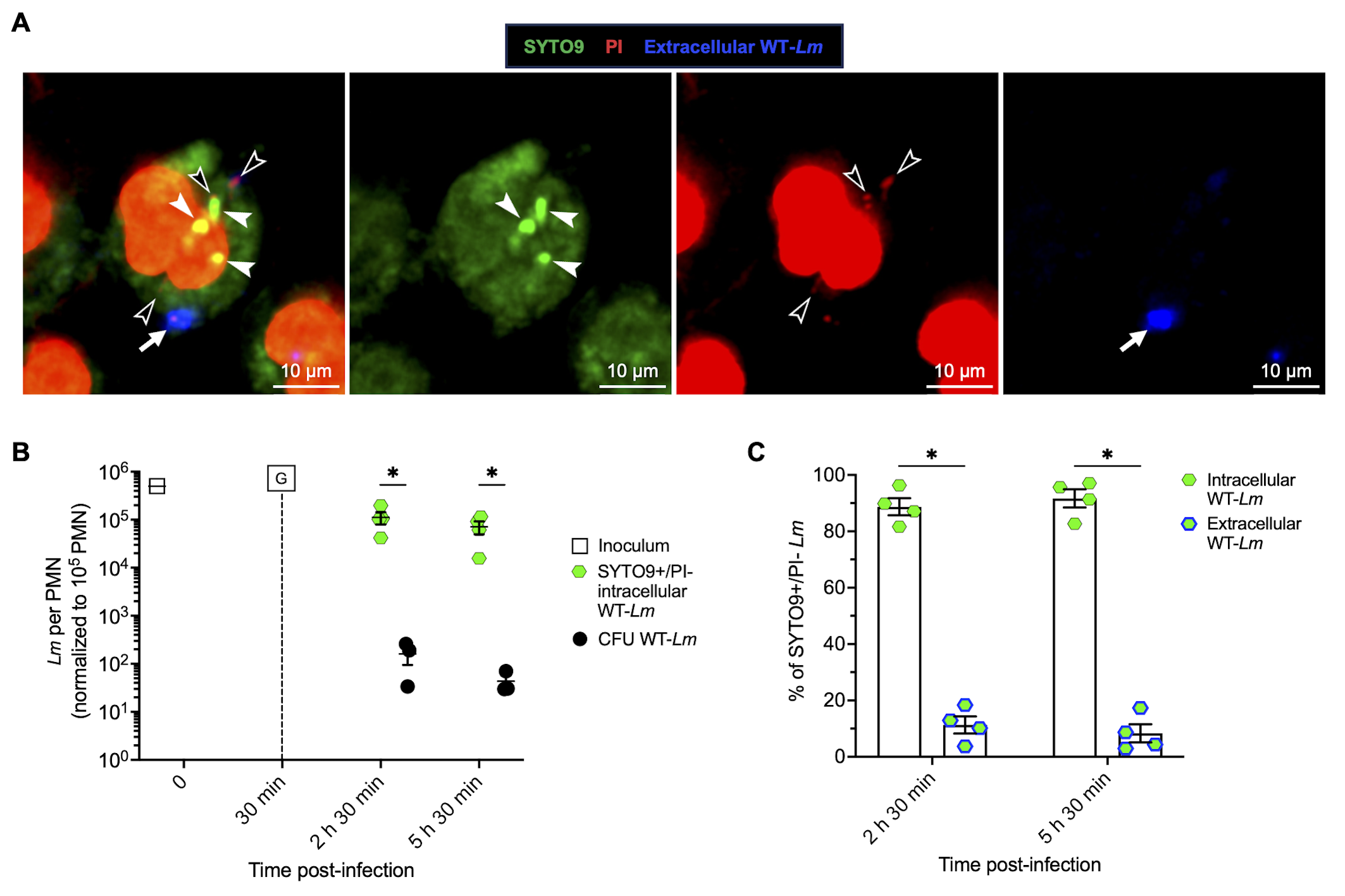

Supplement: Fig. S3 — Lm persist as VBNC forms in human PMNs. [file mbio.02700-24-s0003.tiff]

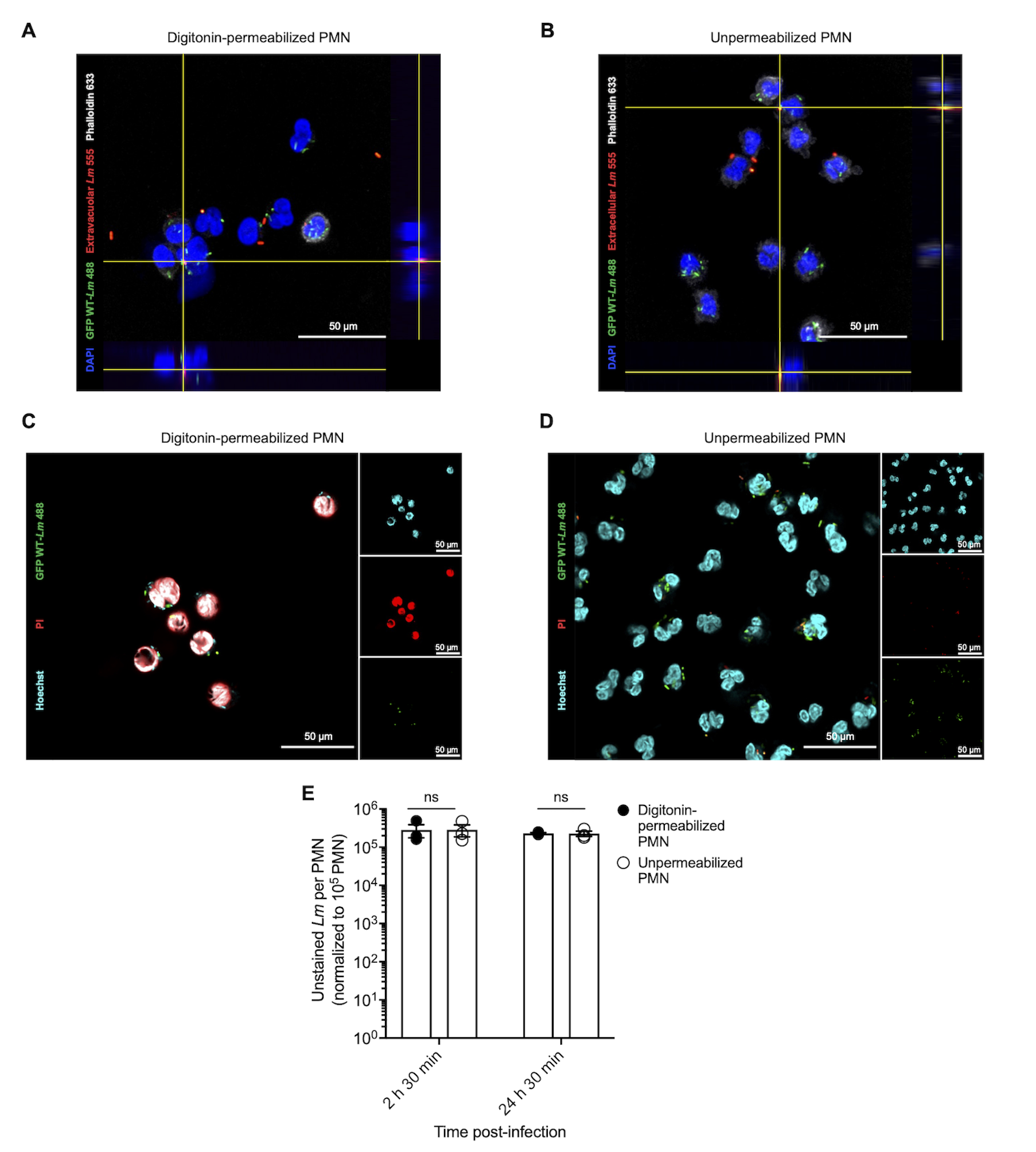

Supplement: Fig. S4 — Lm localization in bovine PMN vacuoles is confirmed by selective digitonin permeabilization. [file mbio.02700-24-s0004.tiff]

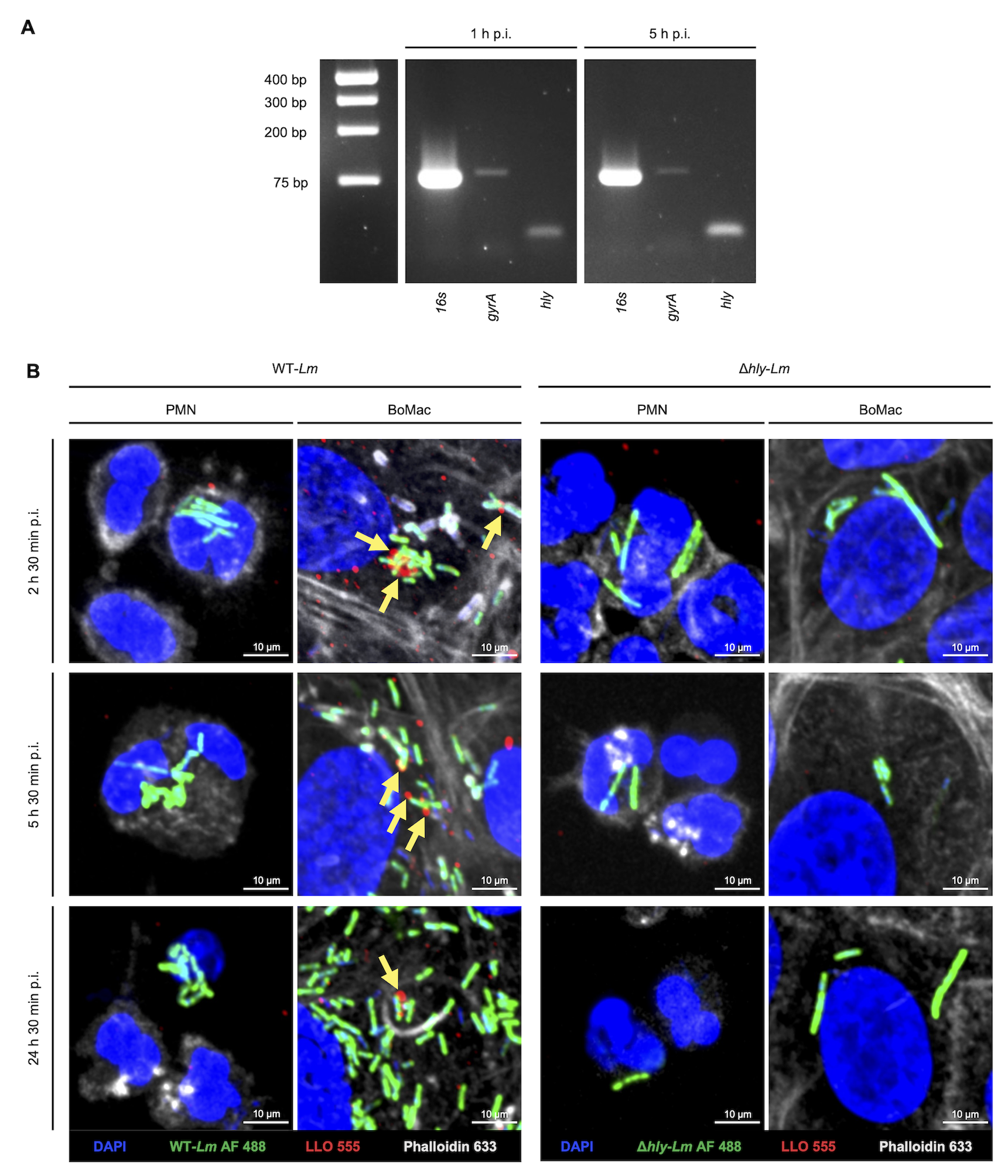

Supplement: Fig. S5 — The hly transcript is expressed by intraneutrophilic Lm without concomitant LLO protein detection by immunofluorescence. [file mbio.02700-24-s0005.tiff]

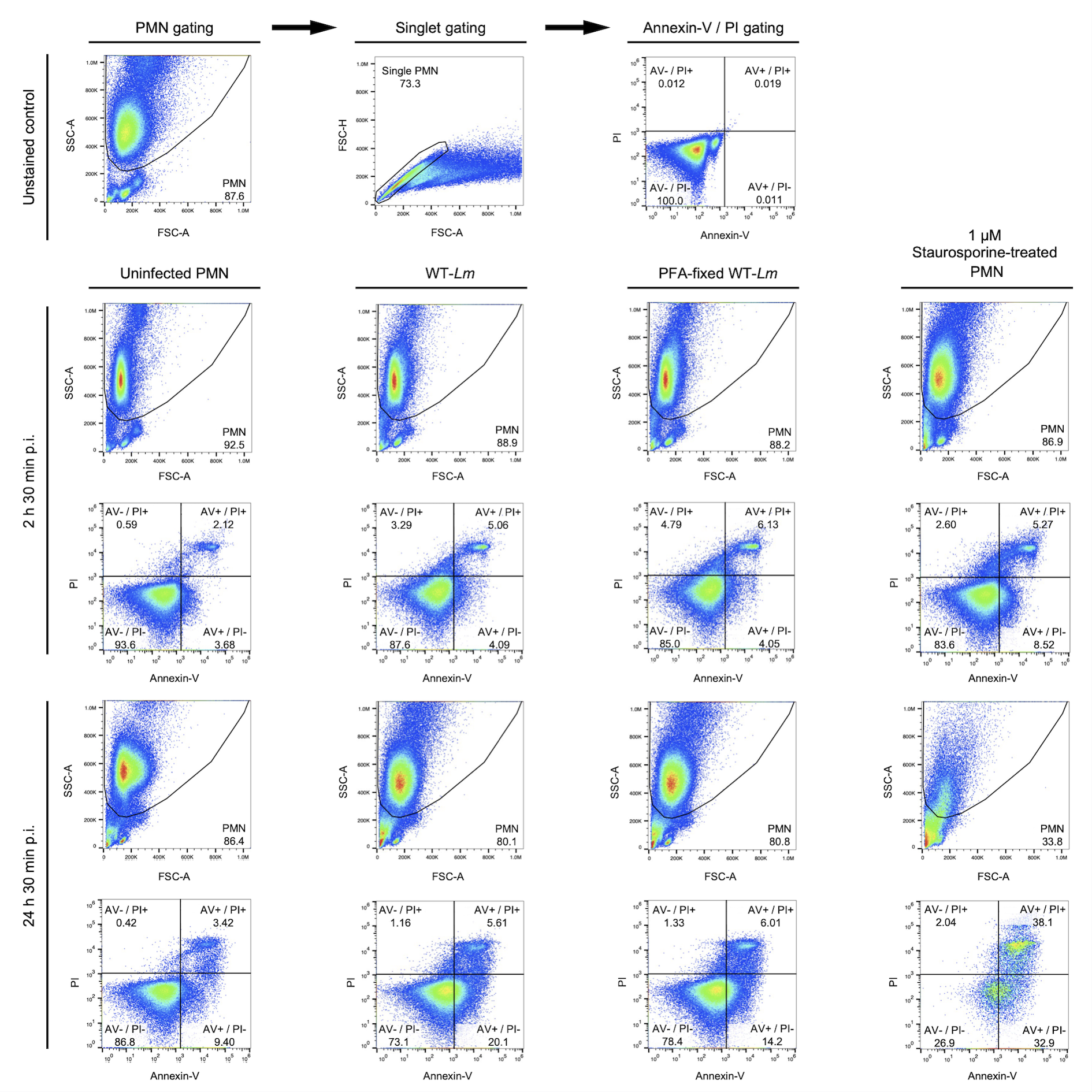

Supplement: Fig. S6 — PMN gating strategy and analysis of bovine PMNs viability after infection with WT-Lm. [file mbio.02700-24-s0006.tiff]

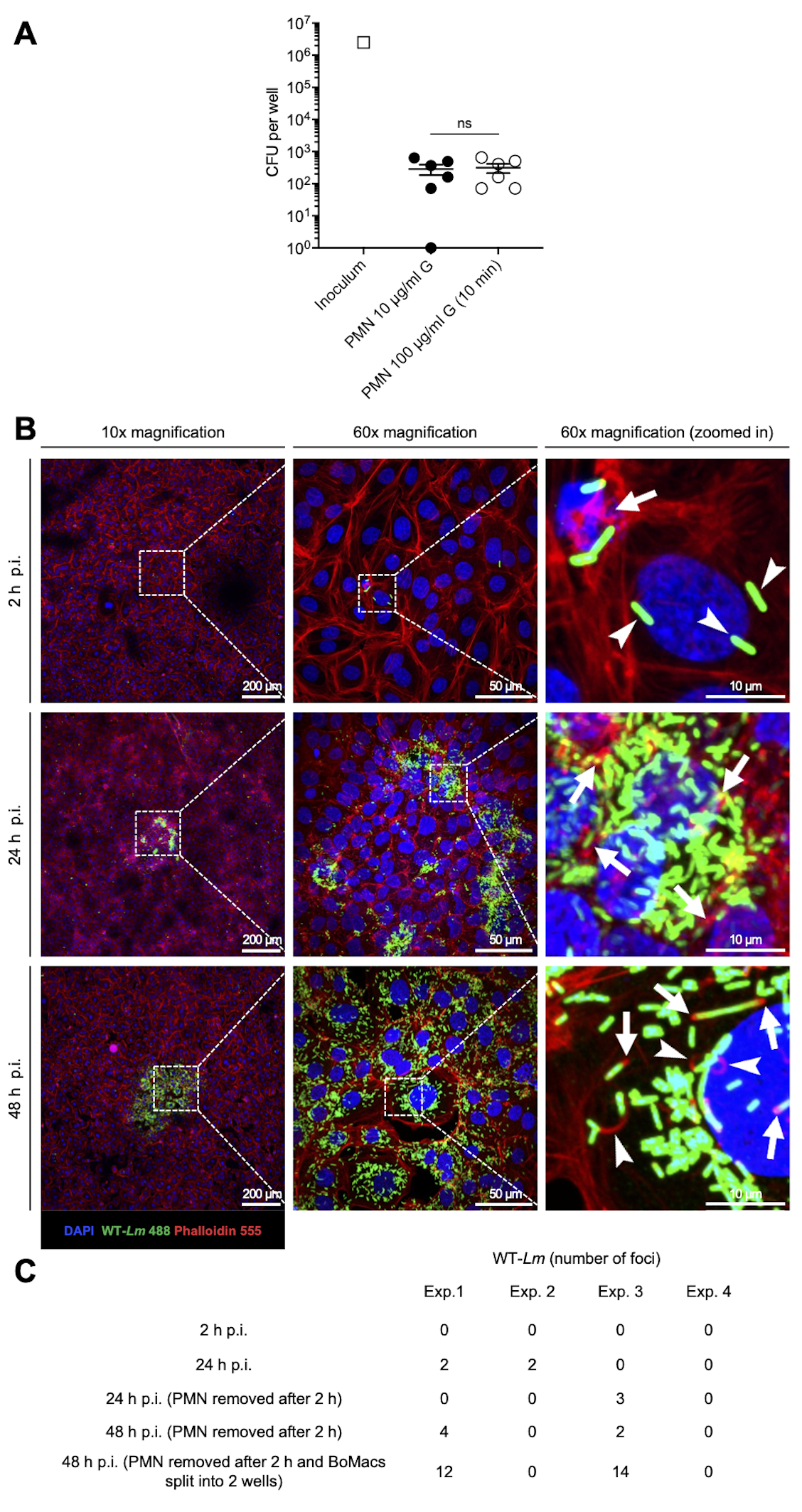

Supplement: Fig. S7 — Lm transmitted from PMNs to BoMacs can establish infectious foci and regain the ability to spread from cell to cell. [file mbio.02700-24-s0007.tiff]

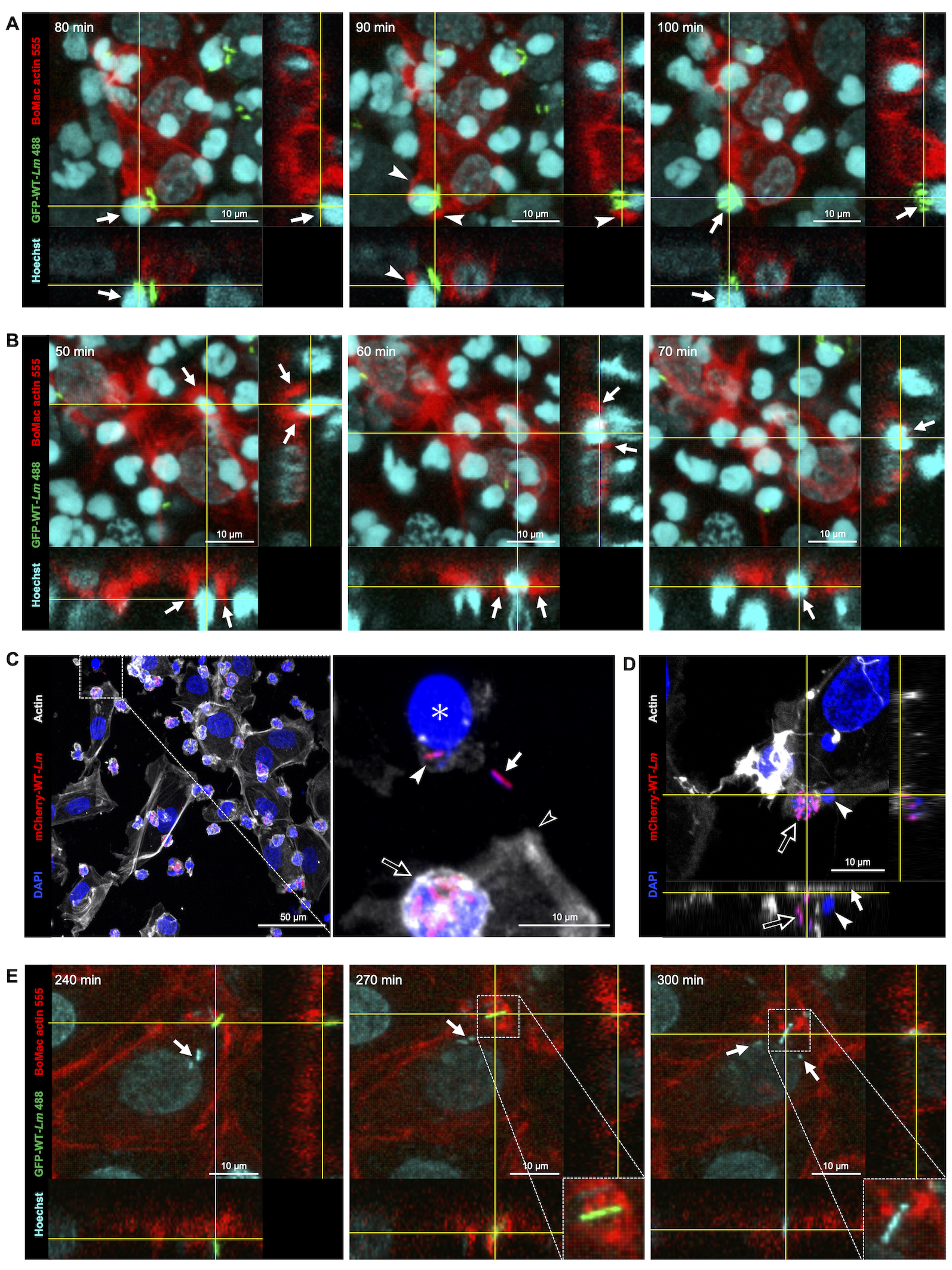

Supplement: Fig. S8 — Representative images of BoMac interactions with Lm-infected PMNs. [file mbio.02700-24-s0008.tiff]
