# Supplementary material for: Intravacuolar persistence in neutrophils facilitates Listeria monocytogenes spread to co-cultured cells
Source: mBio. 2025 Mar 11;16(4):e02700-24. doi: 10.1128/mbio.02700-24 (PMC11980584; doi:10.1128/mbio.02700-24)
Supplement: Legends — for supplemental figures and video. [file mbio.02700-24-s0009.docx]

**SUPPLEMENTARY** **FIGURE LEGENDS, TABLES AND VIDEO LEGENDS**

**Fig. S1.** Bovine PMNs fail to efficiently sterilize extracellular *Lm*. (**A, B**) Killing assay comparing total CFU of WT-*Lm* (**A**: MOI 0.1; **B**: MOI 5) incubated with PMNs (black) and WT-*Lm* incubated without PMNs (white). (**C**) Percentage of bacterial killing at 10 min and 30 min p.i. in the killing assay (MOI 0.1: black; MOI 5: white). Values indicate CFU reduction of WT-*Lm* incubated with PMNs compared to bacteria incubated without PMNs. (**D**) Killing assay comparing non-opsonized WT-*Lm* (black) to serum-opsonized WT-*Lm* (grey), both incubated with PMNs at MOI of 5. Depending on the MOI used, CFU were normalized to an initial inoculum of 10^4^ CFU (MOI 0.1: **A**) or 5x10^5^ CFU (MOI 5: **B, D**). Data are expressed as the mean (± SEM) of CFU per well from 3 independent experiments performed in triplicate. Data were analyzed by Kruskal-Wallis test (**A, B, D**). * P = < 0.05; ** = P < 0.01; *** = P < 0.001; **** = P < 0.0001; ns = not significant.

**Fig. S2.** Serum opsonization has a moderate to no effect on PMN listericidal efficacy and on *Lm* fitness. (**A**) Killing assay comparing serum-opsonized WT-*Lm* (grey, from Fig. S1D) incubated with PMNs with opsonized WT-*Lm* incubated without PMNs (white). (**B**) Gentamicin protection assay of serum-opsonized WT-*Lm* incubated with PMNs (grey, from Fig. 1D). CFU numbers are compared to the first incubation time point (40 min p.i.). (**C**) Non-opsonized WT-*Lm* (black empty circles) compared to serum-opsonized WT-*Lm* (grey empty circles, from (**A**)), both incubated in medium in the absence of PMNs. (**D**) Comparison between opsonized GFP+ WT-*Lm* (grey diamonds), serum-opsonized viable intracellular WT-*Lm* (green hexagons with grey borders), and CFU of opsonized WT-*Lm* (grey circles). Data are obtained from 3 independent experiments performed in triplicate (CFU), or from 5 (SYTO9+/PI- intracellular *Lm*) and 10 (GFP+ bacteria) independent FOVs per replicate and time point. CFU data are from Fig. 5A, as these experiments were performed in parallel with those that generated the GFP+ *Lm* data illustrated here. (**E**) Comparison between non-opsonized intracellular WT-*Lm* (green, from Fig. 2G) and serum opsonized intracellular WT-*Lm* (green with grey borders, from (**D**)). 5 independent FOVs were analyzed per replicate and time point. (**F**) Percentage of non-opsonized GFP+ WT-*Lm* (black) and serum-opsonized GFP+ WT-*Lm* (grey) associated with LAMP-1**.** Ten independent FOVs were analyzed per replicate and each time point. (G) Gentamicin protection assay comparing non-opsonized Δ*hly*-*Lm* (grey, from Fig. 5A) with serum-opsonized Δ*hly*-*Lm* (white circles with grey border), both incubated with PMNs. Data are expressed as mean (± SEM) of CFU per well from 3 independent experiments performed in triplicate, with CFU normalized to an initial inoculum of 5x10^5^ CFU. (**H**) Gentamicin protection assay comparing non-opsonized Δ*actA*-*Lm* (white circles with black borders, from Fig. 5A) with serum-opsonized Δ*actA*-*Lm* (white circles with grey borders), both incubated with PMNs. Data are expressed as mean (± SEM) of CFU per well from 3 independent experiments performed in triplicate, with CFU normalized to an initial inoculum of 5x10^5^ CFU. (**I**) Gentamicin protection assay comparing serum-opsonized GFP-expressing WT-*Lm* (black) with serum-opsonized GFP Δ*hly*-*Lm* (grey, from (**G**)) or Δ*actA*-*Lm* (white, from (**H**)). For each time point, CFU of the WT are compared to both Δ*hly* and Δ*actA* deletion mutants. (**J**) Number of GFP+ intraneutrophilic serum-opsonized WT-*Lm* (black, from (**D**)), Δ*hly*-*Lm* (grey) and Δ*actA*-*Lm* (white). 10 independent FOVs were analyzed per replicate and time point. For each time point, the number of opsonized GFP+ WT-*Lm* is compared to both Δ*hly* and Δ*actA* deletion mutants. (**K**) Percentage of GFP+ WT-*Lm* (black, from (**F**)), Δ*hly*-*Lm* (grey) and Δ*actA*-*Lm* (white) in association with LAMP-1**.** 10 independent FOVs were analyzed per replicate and time point. Data are expressed as mean (± SEM) of: I) CFU per well from 3 independent experiments performed in triplicate, with CFU normalized to an initial inoculum of 5x10^5^ CFU (**A-C, G-I**), II) *Lm* per 10^5^ PMNs from 3 independent experiments (**D, E, J**), or III) percentage from 3 independent experiments (**F, K**). Where indicated, gentamicin was added to the medium at 30 min p.i. (G). Data were analyzed by Kruskal-Wallis test (**A, C, E-K**) or one-way ANOVA followed by planned comparisons (**B, D**). * = P < 0.05; ** = P < 0.01; **** = P < 0.0001; ns = not significant.

**Fig. S3.** *Lm* persist as VBNC forms in human PMNs**.** (**A**) Representative MAX intensity projection of a Z-stack from a BacLight viability assay combined with differential intra-/extracellular *Lm* staining of human PMNs infected with *Lm* (2 h 30 min p.i.). Viable *Lm* are shown in green (white arrowheads), dead *Lm* are represented in red (empty arrowheads), and extracellular *Lm* are represented in blue (white arrow). (**B**) Comparison between viable intracellular *Lm* (light green) and CFU (black) in human PMNs. Data are expressed as mean (± SEM) of *Lm* per 10^5^ PMNs from 3 independent experiments, in which 5 independent FOVs were analyzed per replicate and time point (for SYTO9+/PI- intracellular WT-*Lm*) or CFU of triplicates were counted, with CFU normalized to an initial inoculum of 5x10^5^ CFU. Gentamicin (G) was added to the medium at 30 min p.i.. (**C**) Percentage of PMN-associated intracellular viable WT-*Lm* (green) compared to PMN-associated extracellular viable bacteria (green with blue border). Data are expressed as mean (± SEM) from 3 independent experiments (in which 5 independent FOVs were analyzed per replicate and time point). Data were analyzed by Kruskal-Wallis test (**B, C**). * = P < 0.05.

**Fig. S4.** *Lm* localization in bovine PMN vacuoles is confirmed by selective digitonin permeabilization. (**A, B**) Representative images of MAX intensity- and orthogonal projections from Z-stacks of digitonin-permeabilized (**A**) and non-permeabilized (**B**) PMNs incubated with GFP WT-*Lm* for 2 h 30 min p.i.. Extravacuolar bacteria are shown in red, while intravacuolar bacteria are shown in green or blue. Due to the small size of PMNs, even in orthogonal views it is not possible to distinguish whether extravacuolar bacteria (red) are located in the cytosol after vacuolar escape or adhere extracellularly to the PMN membrane (i.e., on the inner or outer side of the phalloidin ring, respectively). Therefore, the data shown in (**E**) were obtained by comparing the number of intravacuolar bacteria (green, blue) between permeabilized and non-permeabilized PMNs. (**C, D**) Representative images of positive controls for PMN permeabilization with digitonin (**C**) and for non-permeabilized PMNs (**D**) incubated with GFP WT-*Lm* for 2 h 30 min p.i. Note how all PMNs nuclei in (**C**) are stained red with PI, indicating an efficient permeabilization of the cell membrane**.** On the other hand, all nuclei of unpermeabilized PMNs do not stain with PI, indicating intact cell membranes (**D**). (**E**) The number of intravacuolar WT-*Lm* in digitonin-permeabilized PMNs (black) is almost equal to the number of intracellular WT-*Lm* in non-permeabilized PMNs (white), indicating that essentially all intracellular bacteria are located within vacuoles. These data are in line with the lack of actin-polymerisation around bacteria. Data are expressed as mean (± SEM) of *Lm* per 10^5^ PMNs from 3 independent experiments (in which 10 independent FOVs were analyzed per replicate and time point). Data were analyzed by Kruskal-Wallis test. ns = not significant.

**Fig. S5.** The *hly* transcript is expressed by intraneutrophilic *Lm* without concomitant LLO protein detection by immunofluorescence. (**A**) RT-PCR reveals *hly* RNA expression at 1 h and 5 h p.i. Bacterial house-keeping genes *16s* and *gyrA* are also shown. Representative images from one of two independent experiments. (**B**) LLO (red) is expressed in BoMacs infected with WT-*Lm* and co-localizes with bacteria at all time points (yellow arrows) while no LLO is detected in association with intraneutrophilic *Lm*. No LLO immunolabeling is detected in BoMacs and PMNs infected with Δ*hly*-*Lm* (negative control), demonstrating the specificity of the LLO antibody used. Representative MAX intensity projections from one of two independent experiments.

**Fig. S6.** PMN gating strategy and analysis of bovine PMNs viability after infection with WT-*Lm*. Representative FACS plots from one experiment (for reasons of space and simplicity, plots from 5 h 30 min p.i. have been omitted). As shown for the unstained control, PMNs are first gated based on their FSC-A vs SSC-A localization, then singlets are gated on an FSC-A vs FSC-H plot and, finally, the gating for Annexin-V (AV) vs PI is determined based on the unstained control sample plot. The PMN gating strategy used for the unstained control was also applied to all other conditions tested (for these conditions, the singlet gating plots have not been included in this figure for simplicity). Note that, with the exception of Staurosporine-treated cells (in which the number of events outside of PMN gating shifts toward the lower corner of the plot, indicating increased cell debris and, thus, PMN lysis), PMN numbers do not decrease drastically over time, indicating that bovine PMNs remain largely intact during prolonged culture and upon infection with/phagocytosis of *Lm*.

**Fig. S7.** *Lm* transmitted from PMNs to BoMacs can establish infectious foci and regain the ability to spread from cell to cell. (**A**) CFU of PMNs incubated with WT-*Lm* for 24 h 30 min in medium containing 10 µg/ml gentamicin (PMN 10 µg/ml G) compared to those of PMNs incubated with WT-*Lm* for 24 h 30 min in medium containing 10 µg/ml gentamicin and further exposed to 100 µg/ml gentamicin for 10 min (PMN 100 µg/ml G (10 min)). CFU are normalized to an initial inoculum of 2.5x10^6^ CFU. Data are expressed as mean ± SEM of pooled triplicates from 2 independent experiments. Data were analyzed by Kruskal-Wallis test. ns = not significant. (**B**) Confocal microscopy of *Lm* in BoMacs after PMN removal (representative images from 1 of 4 independent experiments, MAX intensity projections). At 2 h p.i. BoMacs contain only few *Lm* without polymerized actin (arrowheads). Note the *Lm* associated with a PMN that was not removed by repeated washing (arrow). At 24 h p.i. *Lm* have formed small infectious foci that span adjacent BoMacs, in which *Lm* are surrounded by actin polymerizations (arrows). At 48 h p.i., larger infectious foci are observed, in which *Lm* frequently polymerize actin as polar actin clouds (arrows) and as propulsive actin tails (arrowheads). (**C**) Number of *Lm* foci observed in 4 experiments. PMNs were co-cultured with BoMacs for 2 or 24 h before removal for counting of BoMacs CFU (2 h p.i. and 24 h p.i., respectively), or PMNs were removed after co-culture with BoMacs for 2 h and BoMacs were further cultured for 24 or 48 h before CFU quantification. In the last setup, BoMacs were not grown to overconfluence but were sub-cultured into two wells at 24 h (the number of foci here refers to the sum of foci observed in both wells).

**Fig. S8.** Representative images of BoMac interactions with *Lm*-infected PMNs. (**A**) A *Lm*-containing PMN in close proximity to a BoMac (80 min, left panel, arrow) is contacted by BoMac actin protrusions (90 min, middle panel, arrowheads) which then retract without internalizing the PMN (probing behavior) (100 min, right panel, arrows). (**B**) Live-cell imaging sequences showing a PMN being contacted by cup-shaped BoMac actin protrusions (arrows, left panel, 50 min of co-culture). The PMN is gradually internalized (arrows, middle panel, 60 min) until it is completely sealed inside the BoMac (arrows, right panel, 70 min). (**C**) An extracellular mCherry+ *Lm* (white arrow) is observed in close proximity to a dying PMN (characterized by dissolving actin and a round, shrunken nucleus; asterisk)(compare with a PMN with an intact membrane containing numerous *Lm*; empty arrow) containing other mCherry+ and mCherry- phagocytosed *Lm* (white arrowhead), and appears to be orienting toward a BoMac in the process of forming an actin protrusion (empty arrowhead). Representative image at 2 h of co-culture. (**D**) Remnants of a dead/apoptotic PMN, consisting of a cluster of both mCherry+ and mCherry- *Lm* (empty arrow), and an associated nuclear fragment compatible with an apoptotic body (white arrowhead), in close contact with a BoMac. Orthogonal views are centered on an mCherry+ *Lm* from such a cluster, which appears in the process of entering the BoMac membrane (white arrow). Representative image at 24 h of co-culture. (**E**) An extracellular GFP+-*Lm* (240 min, left panel) is surrounded by BoMac actin protrusions (270 min, middle panel; inset), leading to its internalization and loss of GFP signal (300 min, right panel; inset). Note that the BoMac contains already GFP- bacteria at 240 min (arrows). (**A, B, E**) STD projections with orthogonal views. (**C, D**) MAX intensity projections with orthogonal views.

**Video S1.** Collated movies of live-cell imaging depicting the whole recorded sequences illustrated in the manuscript’s Figures. Please refer to these figures for details regarding the events illustrated. The sequences in Fig. 6E (30-105 min) show that a *Lm*-containing PMN interacts closely with a BoMac cell (slightly above the center of the images). From 60 min onwards, BoMac actin protrusions envelop the PMN and its associated bacteria (one of which displays loss of GFP). Sequences referring to Fig. 6F (30-110 min) depict a group of GFP-expressing *Lm* being contacted and internalized by BoMac actin protrusions (slightly left of the center of the images) from 40 min onwards, with one of such bacteria displaying loss of GFP. Sequences referring to Fig. S8A (30-110 min) show a BoMac contacting a PMN (lower left quadrant of the images) through actin cups (from 40 min onwards) and protrusions without internalization of the PMN. Sequences referring to Fig. S8B (30-110 min) depict a PMN devoid of associated *Lm* (upper right quadrant of the images) being contacted by actin cups and protrusions, which ultimately results in its internalization (from 60 min onwards). In Fig. S8E (240-345 min), an extracellular GFP-positive *Lm* (upper right quadrant of the images) is contacted and internalized by BoMac actin protrusions from 270 min onwards, resulting in loss of GFP upon internalization from 300 min onwards.
